# Supplementary material for: EnDPoINT: protocol for an implementation research study to integrate a holistic package of physical health, mental health and psychosocial care for podoconiosis, lymphatic filariasis and leprosy into routine health services in Ethiopia
Source: BMJ Open. 2020 Oct 15;10(10):e037675. doi: 10.1136/bmjopen-2020-037675 (PMC7566734; doi:10.1136/bmjopen-2020-037675)
Supplement: Author's manuscript [file bmjopen-2020-037675.draft_revisions.pdf]

BMJ Open

BMJ Open is committed to open peer review. As part of this commitment we make the peer review history of every article we publish publicly available.

When an article is published we post the peer reviewers' comments and the authors' responses online. We also post the versions of the paper that were used during peer review. These are the versions that the peer review comments apply to.

The versions of the paper that follow are the versions that were submitted during the peer review process. They are not the versions of record or the final published versions. They should not be cited or distributed as the published version of this manuscript.

BMJ Open is an open access journal and the full, final, typeset and author-corrected version of record of the manuscript is available on our site with no access controls, subscription charges or pay-per-view fees (http://bmjopen.bmj.com).

If you have any questions on BMJ Open's open peer review process please email info.bmjopen@bmj.com

BMJ Open

EnDPoINT – Protocol for an implementation research study to integrate a holistic package of physical health, mental health and psychosocial care for podoconiosis, lymphatic filariasis and leprosy into routine health services in Ethiopia

Journal:	*BMJ Open*
Manuscript ID	bmjopen-2020-037675
Article Type:	Protocol
Date Submitted by the Author:	11-Feb-2020

Complete List of Authors:	Semrau, Maya; Brighton and Sussex Medical School, Centre for Global Health Research<br>Ahmed, Oumer; Brighton and Sussex Medical School, Centre for Global Health Research; Addis Ababa University, Center for Innovative Drug Development and Therapeutic Trials for Africa (CDT-Africa)<br>Deribe, Kebede; Brighton and Sussex Medical School, Centre for Global Health Research; Addis Ababa University, School of Public Health, College of Health Sciences<br>Mengiste, Asrat; Addis Ababa University, Center for Innovative Drug Development and Therapeutic Trials for Africa (CDT-Africa)<br>Tesfaye, Abraham; Addis Ababa University, Center for Innovative Drug Development and Therapeutic Trials for Africa (CDT-Africa)<br>Kinfe, Mersha; Addis Ababa University, Center for Innovative Drug Development and Therapeutic Trials for Africa (CDT-Africa)<br>Bremner, Stephen; Brighton and Sussex Medical School, Department of Primary Care and Public Health<br>Hounsome, Natalia; Brighton and Sussex Medical School, Centre for Global Health Research<br>Kelly-Hope, Louise; Liverpool School of Tropical Medicine, Centre for Neglected Tropical Diseases, Department of Tropical Disease Biology<br>MacGregor, Hayley ; Institute of Development Studies<br>Taddese, Henock; Imperial College London, Faculty of Medicine, School of Public Health<br>Banteyerga, Hailom ; Addis Ababa University, College of Humanities, Language Studies, Journalism and Communication<br>HaileMariam, Damen; Addis Ababa University, School of Public Health, College of Health Sciences<br>Negussu, Nebiyu; Federal Ministry of Health<br>Fekadu, Abebaw; Brighton and Sussex Medical School, Centre for Global Health Research; Addis Ababa University, Center for Innovative Drug Development and Therapeutic Trials for Africa (CDT-Africa)<br>Davey, Gail; Brighton and Sussex Medical School, Centre for Global Health Research

Keywords:	MENTAL HEALTH, INFECTIOUS DISEASES, PRIMARY CARE, PUBLIC HEALTH

I, the Submitting Author has the right to grant and does grant on behalf of all authors of the Work (as defined in the below author licence), an exclusive licence and/or a non-exclusive licence for contributions from authors who are: i) UK Crown employees; ii) where BMJ has agreed a CC-BY licence shall apply, and/or iii) in accordance with the terms applicable for US Federal Government officers or employees acting as part of their official duties; on a worldwide, perpetual, irrevocable, royalty-free basis to BMJ Publishing Group Ltd ("BMJ") its licensees and where the relevant Journal is co-owned by BMJ to the co-owners of the Journal, to publish the Work in this journal and any other BMJ products and to exploit all rights, as set out in our licence.

The Submitting Author accepts and understands that any supply made under these terms is made by BMJ to the Submitting Author unless you are acting as an employee on behalf of your employer or a postgraduate student of an affiliated institution which is paying any applicable article publishing charge ("APC") for Open Access articles. Where the Submitting Author wishes to make the Work available on an Open Access basis (and intends to pay the relevant APC), the terms of reuse of such Open Access shall be governed by a Creative Commons licence – details of these licences and which Creative Commons licence will apply to this Work are set out in our licence referred to above.

Other than as permitted in any relevant BMJ Author's Self Archiving Policies, I confirm this Work has not been accepted for publication elsewhere, is not being considered for publication elsewhere and does not duplicate material already published. I confirm all authors consent to publication of this Work and authorise the granting of this licence.

For peer review only

EnDPoINT – Protocol for an implementation research study to integrate a holistic package of physical health, mental health and psychosocial care for podoconiosis, lymphatic filariasis and leprosy into routine health services in Ethiopia

Semrau M[1], Ahmed OA[1,2], Deribe K[1,3], Mengiste A[2], Tesfaye A[2], Kinfe M[2], Bremner SA[4], Hounsome N[1], Kelly-Hope LA[5], MacGregor H[6], Taddese HB[7], Banteyerga H[8], HaileMariam D[3], Negussu N[9], Fekadu A[1,2*], Davey G[1*]

[1] Centre for Global Health Research, Brighton and Sussex Medical School, Brighton, United Kingdom

[2] Center for Innovative Drug Development and Therapeutic Trials for Africa (CDT-Africa), Addis Ababa University, Addis Ababa, Ethiopia

[3] School of Public Health, College of Health Sciences, Addis Ababa University, Addis Ababa, Ethiopia

[4] Department of Primary Care and Public Health, Brighton and Sussex Medical School, Brighton, United Kingdom

[5] Centre for Neglected Tropical Diseases, Department of Tropical Disease Biology, Liverpool School of Tropical Medicine, Liverpool, United Kingdom

[6] Institute of Development Studies, Brighton, United Kingdom

[7] Faculty of Medicine, School of Public Health, Imperial College London, London, United Kingdom

[8] College of Humanities, Language Studies, Journalism and Communication; Addis Ababa University, Addis Ababa, Ethiopia

[9] Federal Ministry of Health, Addis Ababa, Ethiopia

[*] joint last authors

Corresponding author: Dr Maya Semrau, Global Health and Infection Department, Room G19, Medical Research Building, Brighton and Sussex Medical School, University of Sussex, Falmer, Brighton, BN1 9PS, United Kingdom; email: m.semrau@bsms.ac.uk; phone: +44-1273-872788

Word count: 4126

For peer review only - http://bmjopen.bmj.com/site/about/guidelines.xhtml

ABSTRACT

Introduction: Neglected tropical diseases (NTDs) causing lower-limb lymphoedema such as podoconiosis, lymphatic filariasis (LF) and leprosy are common in Ethiopia. Routine health services for morbidity management and disability prevention (MMDP) of these conditions are still lacking, even though they impose a huge burden on affected individuals and their communities in terms of physical and mental health, and psychosocial and economic outcomes. This calls for an integrated, holistic approach to MMDP across these three diseases.

Methods and analysis: The 'Excellence in Disability Prevention Integrated across NTDs' (EnDPoINT) implementation research study aims to assess the integration and scale-up of a holistic package of care – including physical health, mental health, and psychosocial care – into routine health services for patients with podoconiosis, LF and leprosy in selected districts in Awi zone in the North-West of Ethiopia. The study is being carried out over three phases using a wide range of mixed methodologies. Phase 1 involves the development of a comprehensive holistic care package and strategies for its integration into the routine health services across the three diseases. Phase 2 involves a pilot study conducted in one sub-district in Awi zone, to examine the factors that influence integration and the roles of key health system actors, and to establish the care package's adoption, feasibility, acceptability, fidelity, potential effectiveness, its readiness for scale-up, costs of the interventions, and the suitability of the training and training materials. Phase 3 involves scale-up of the care package in three whole districts, as well as its evaluation in regard to coverage, implementation, clinical (physical health, mental health and psychosocial), and economic outcomes.

Ethics and dissemination: Ethics approval for the study has been obtained in the UK and Ethiopia. The results will be disseminated through publications in scientific journals, conference presentations, policy briefs and workshops.

Key words: lymphoedema, podoconiosis, lymphatic filariasis, leprosy, mental health, implementation research

For peer review only - http://bmjopen.bmj.com/site/about/guidelines.xhtml

ARTICLE SUMMARY

Strengths and limitations of this study

- The EnDPoINT implementation research study takes a holistic approach in that it explores the full scope of processes and outcomes involved in the integration of physical health, mental health, and psychosocial care for people with podoconiosis, lymphatic filariasis (LF) and leprosy (including interventions to ensure reduction of stigma towards people with these diseases), both in regards to integration of care into the routine health services in Ethiopia as well as integration of holistic care across the three conditions.
- EnDPoINT is guided by relevant conceptual perspectives on implementation research as well as employing a 'Theory of Change' (ToC) approach towards devising, managing and evaluating the integration study.
- The study uses a wide range of mixed-methods approaches across three phases to develop, scale-up and evaluate the holistic care package.
- Limitations of the study are: selection of study districts partly based on accessibility; time and resource constraints that do not allow the pilot study to have a control group or full-length follow-up before the scale-up; and possible contamination of the study results by previous or ongoing work of other organisations in the study zone.
- The study team comprises an array of multi-disciplinary academics and professionals in Ethiopia as well as internationally, including medics, epidemiologists, psychiatrists, policy experts, social scientists and participatory intervention methodologists.

For peer review only

For peer review only - http://bmjopen.bmj.com/site/about/guidelines.xhtml

INTRODUCTION

Neglected tropical diseases (NTDs) that result in lower limb lymphoedema, that is, swelling of the lower leg, include podoconiosis, lymphatic filariasis (LF) and leprosy. These conditions are common in Ethiopia, with an estimated 1.53 million cases of podoconiosis in the country (1), and around 300,000 people affected by leprosy (2). The exact prevalence for LF lymphoedema in Ethiopia is less well known, since recent mapping studies have resulted in differing estimates (3, 4); however integrated morbidity mapping in 2018 in 20 podoconiosis-LF co-endemic districts identified a prevalence of 84.9 per 10,000 population (26,123 cases in total), of whom 95.3% had leg lymphoedema only, 2.9% had hydrocele (swelling of the genitals in men), 1.5% had both leg lymphoedema and hydrocele, and 0.3% cases had breast lymphoedema (5). Nationwide mapping in 2013 demonstrated that podoconiosis accounts for approximately 64.8%, LF for 13.2%, and leprosy for 12.8% of the total burden of lymphoedema in Ethiopia (6). With global burden estimates of 15 million, 4 million and 2-3 million for LF lymphoedema, podoconiosis and people affected by leprosy respectively (7-9), improving morbidity management and disability prevention (MMDP), and enhancing our understanding of processes and outcomes of these efforts, in particular how to integrate MMDP into existing routine primary community-based health care services, are likely to be of global significance and address resource and sustainability questions (10).

Lymphoedema imposes huge burdens on affected individuals and their communities in terms of disability such as reduced mobility and pain (11), mental distress (12), depression and anxiety (13, 14), stigma, discrimination and social exclusion (11, 15-18), which can limit health-seeking behaviours and access to social services including education, thereby leading to loss of economic productivity at household, community and national level (19-22). For podoconiosis in Ethiopia alone, affected people lose on average 45% of their economically productive time due to morbidity associated with the disease (20), and the estimated cost to Ethiopia's economy is $213 million per year (23). These conditions therefore compromise the livelihoods and wellbeing of populations that are already disadvantaged and hard-to-reach, for example because they live remotely (24). Burden of disease measures are even higher when taking into account the psychological and emotional consequences of NTDs, with estimates that the burden of these psychosocial outcomes may be double that of the physical health consequences (25).

The World Health Organization (WHO) has published targets for elimination of many NTDs, including LF and leprosy (26, 27); however, this has not included podoconiosis (though podoconiosis has been included under LF for MMDP). Whilst foot care for leprosy patients has been integrated into routine health services in Ethiopia since 2001 (28), foot care interventions for podoconiosis and LF aiming to prevent disability are currently mainly provided through donor-supported projects in a disease-specific and disparate manner. Knowledge of these neglected diseases within health care systems is therefore often inadequate, diagnosis and treatment options are limited, and the cost of accessing health care to ease symptoms can be prohibitively expensive. On the other hand, the Ethiopian Federal Ministry of Health (FMOH) has developed integrated guidelines for LF and podoconiosis MMDP but these did not include leprosy (nor did they include mental health or psychosocial components). Yet, there is a clear rationale for the integration of care across these three conditions because of the shared clinical symptomatology of lower-leg lymphoedema. In addition, as the mental distress and illness that commonly accompanies lymphoedema (12, 13, 29) often goes untreated (30), care can be enhanced further for these patients by adding mental health and psychosocial components to physical health services. Integration of care for lymphoedema can

For peer review only - http://bmjopen.bmj.com/site/about/guidelines.xhtml

therefore include three aspects: i) integration of care across the three diseases; ii) integration of care for these diseases into routine health services; and iii) integration of mental health and psychosocial interventions into holistic packages of care.

The Ethiopian FMOH has recognized the importance of these issues, and as a result, both podoconiosis and LF have now been included in the first two National Master Plans (2013-2015 and 2015/16-2019/20) for integrated control of NTDs (31, 32) and identified as two of eight priority NTDs in the country, whilst at the same time aiming to move away from vertical programmes. There have also been calls to improve the responsiveness of the health system and include mental health and psychosocial care into the MMDP of podoconiosis and other lymphoedema in Ethiopia (12). The Health Extension Programme, rolled out successfully in Ethiopia since 2003, now boasts more than 38,000 community-based Health Extension Workers (HEWs), and a supervisory system to support them (33). Their reach has been extended through the women-centred Health Development Army (HDA), members of which link 'model families' with five other households to implement health initiatives (34). These cadres are ideally placed to offer simple, low-tech foot care as well as mental health and psychosocial support to people living with lymphoedema in Ethiopia (35), and to refer on patients who need more specialised care.

To address these issues and make use of the opportunities that currently exist in Ethiopia, the FMOH requested implementation research to guide integration of a holistic care package, including physical health, mental health and psychosocial care interventions, for patients with lower-limb lymphoedema into government-run health services. The 'Excellence in Disability Prevention Integrated across NTDs' (EnDPoINT) research study (2018-2021), funded by the National Institute for Health Research (NIHR) in the UK, was set up in response to this and our findings are expected to inform existing plans for scale-up of integrated physical limb care, and mental health and psychosocial support interventions across Ethiopia. The protocol for the EnDPoINT study is outlined below.

METHODS AND ANALYSIS

Conceptual framework for study

EnDPoINT is an implementation research study in that its research questions have a strong focus on implementation strategies, it includes a wide range of implementation measures, and it is being conducted in real-world settings and populations (36). The study takes guidance both from the Context and Implementation of Complex Interventions (CICI) framework, as well as from the Medical Research Council (MRC) framework for development and evaluation of complex interventions (37, 38), with further explication and reinforcement of the different steps within the MRC framework through a 'Theory of Change' (ToC) approach (39).

The CICI is a comprehensive framework that incorporates implementation, context and setting as dimensions, and interventions are seen to be operationalised within the macro, meso and micro levels. A core interest of EnDPoINT is to generate transferable learning as to the mechanisms at work during integration of targeted interventions into health systems within low- and middle-income countries. As such, the study is a theory-led investigation, for which the CICI can provide a useful framework. We hope to generate rich descriptions of processes and mechanisms of effect, within

For peer review only - http://bmjopen.bmj.com/site/about/guidelines.xhtml

the Ethiopian context, thereby enabling inference and transferability to similar settings and conditions.

The MRC framework for complex interventions provides a model for the practical steps involved in the development and evaluation of EnDPoINT's care package (sitting within the broader theoretical framework provided by the CICI). The MRC framework has been used widely and proposes four phases in the development and evaluation of interventions: i) development, ii) feasibility/piloting, iii) evaluation, and iv) implementation; these phases are considered to be an iterative rather than a linear process, and can facilitate locally appropriate strategies to be generated. ToC is a structured thinking process to build a 'causal pathway' of what needs to change and why, in order to achieve the desired outcomes (39); it enhances the MRC framework for complex interventions through its emphasis on a theory-driven approach, which can be incorporated into and provide practical guidance for the different phases of the MRC framework (39). Figure 1 shows how ToC may theoretically link in and strengthen each of the four phases of the MRC framework on complex interventions. Within the EnDPoINT study, the ToC approach will be used within two of the four phases of the MRC framework: the development and feasibility/piloting phases; for these two phases, there is good evidence for the practical viability and usefulness of an embedded ToC approach (40).

Figure 1 around here

Study design

EnDPoINT's aims are to facilitate effective access to physical health, mental health and psychosocial care for patients with podoconiosis, LF and leprosy in selected districts in Awi zone in Ethiopia through integration and scale-up of a holistic care package into government-run health services.

The study is organised according to three phases, which map onto the different phases of the MRC framework on complex interventions (38, 41). It takes an iterative approach, whereby the subsequent phases are dependent on findings made during the previous phases.

Phase 1 of EnDPoINT – corresponding to Phase 1 of the MRC framework – entails the development of the holistic care package, and includes activities to inform the development of the core package of care and strategies for its integration into the routine health care delivery system, as well as integration of MMDP across the three diseases.

Phase 2 – corresponding to Phase 2 of the MRC framework – involves a pilot study of the holistic care package in one sub-district in Awi zone, which includes activities to study the factors that influence integration of the care package into the state health system and the roles of key health system actors, as well as integration of the care package across the three diseases.

Phase 3 – corresponding to Phases 3 and 4 of the MRC framework – entails the scale-up of the holistic care package of the integrated three disease MMDP embedded into the primary health care facilities, and its evaluation in three selected districts in Awi zone.

For peer review only - http://bmjopen.bmj.com/site/about/guidelines.xhtml

See Table 1 for the objectives, research questions, activities and outcome measures for each of the three phases.

Table 1 around here

Sample selection

Awi zone, in which the EnDPoINT study is being conducted, is one of ten zones in the Amhara Region in the North-West of Ethiopia and is divided into twelve districts (or *woredas*). Figure 2 shows the three districts selected for the study: one sub-district in Guagusa Shikudad district for piloting during Phase 2; and three whole districts for the scale-up during Phase 3 (Guagusa Shikudad, Ankesha Guagusa and Guangua). The districts were chosen based on: co-endemicity of podoconiosis, LF and leprosy; any previous or ongoing work in the districts; and accessibility. The population of Awi zone is 982,942 (with each district/*woreda* having populations of between around 8000 and 31,500), according to the latest Ethiopian Census in 2007 (42). As for most of Ethiopia, the zone is largely a rural area (around 87.5% of the population (42)); Agew-Awi (59.8%) and Amhara (38.4%) are the two main ethnic groups, and the two most commonly spoken first languages are Amarigna (53.4%) and Agew-Awigna (45.0%). The large majority of the population (94.4%) are Ethiopian Orthodox Christians (42).

Figure 2 around here

A wide range of stakeholders are involved in the EnDPoINT study. For the qualitative research activities, such as the workshops, key informant interviews and focus groups (see Table 1), purposive sampling techniques are being used to identify, approach and recruit key stakeholders based on their role and position. These include members of the EnDPoINT study consortium who have expertise in the provision of care and the context in Ethiopia, current NTD and/or mental health programme implementers (including non-governmental organisations), personnel in the NTD Department of the Ethiopian FMOH, policy-makers, health service planners, health managers and decision-makers, facility-based health workers (e.g. primary health care staff, health officers, nurses), community health workers, community leaders, traditional and religious leaders, members of service user organisations, and service users and their families. For the situational analysis, key health officials and service managers are being approached based on purposive sampling methods, if necessary, though most of the information collected for this is being based on secondary data that are available in the public domain.

Furthermore, during the pilot study and scale-up of the care package in Phases 2 and 3, key stakeholders will be purposively selected to receive training to implement the various components (interventions) of the care package; this includes staff at the health care organisation level, zonal and district level health office staff, psychiatric nurses, senior health care workers, pharmacy staff, health centre staff (health officers and nurses), community health workers (CHWs) / HEWs, and members of the community. In addition, affected people, i.e. those with podoconiosis, LF and leprosy, as well as their families and communities, are being included in the study; these are people identified as

For peer review only - http://bmjopen.bmj.com/site/about/guidelines.xhtml

requiring care for their lower limb lymphoedema and/or co-morbid mental ill-health. Affected people will be beneficiaries of some interventions (a few of whom will be selected as case studies), but will also potentially contribute to the delivery of some of the training packages and/or interventions, for example by joining training sessions in order to provide a 'lived testimony' of living with the disease; this is because of growing evidence that social contact elements within interventions, whereby people come into contact with affected patients and hear their stories, are most effective in reducing stigma and discrimination, and can contribute to increased service uptake and provision.

Only adults are being included in the study, i.e. participants will all be at least 18 years of age. All components of the EnDPoINT study are being conducted in either Amharic or English, depending on which language is most suitable to the particular participant group. Where necessary, all study instruments and materials, including participant information sheets and consent forms, are translated into Amharic.

Sample size justification

For the qualitative research activities, such as key informant interviews and focus group discussions, the sample size is guided by the number of key stakeholders who are identified to take part. A data saturation approach is being used (i.e. data collection will continue until sufficient information has been obtained or where further data collection fails to generate new/additional themes), which is usual and appropriate for qualitative data collection techniques. For the workshops and training components, the sample size will be established based on the number of people who are identified to be suitable and available. During Phase 2, all health staff in the selected sub-district are trained; during Phase 3, all relevant health staff will be trained in the selected three districts. Similarly, all adult patients who are identified within the single sub-district during Phase 2 and within the three whole districts during Phase 3 as having either podoconiosis, LF or leprosy will receive the care interventions included in the care package (or will at least be offered these). The sample size during any workshops and focus groups will not exceed 16 participants, to ensure that all participants have the opportunity to speak.

Key interventions within the care package

Care package interventions

The EnDPoINT care package is organised according to three levels of the health care system: health care organisation/coordination, health care facility, and community level. This may include the following interventions, though these will be finalised only after completion of Phase 2 due to the iterative nature of the study:

- Health care organisation/coordination:
    - High-level awareness-raising and mobilisation: participatory workshop for zonal and district health bureau officials, and evidence generation for best practice
    - Programme management, i.e. working in partnership with key health care organisation staff to ensure the necessary structures and budget for the delivery of the care package

For peer review only - http://bmjopen.bmj.com/site/about/guidelines.xhtml

  - Capacity-building, i.e. 'Training of Trainers' (ToT) for health care coordination staff about lymphoedema MMDP and mental health care provision, and supportive supervision, mentoring and coaching
- Health facility:
  - Capacity-building for facility-based health centre staff: training on lymphoedema MMDP and mental health care provision, and training on supply chain management for those staff who manage MMDP supplies
  - Awareness-raising and stigma reduction: i) participatory workshop for facility-based staff; ii) posters in health centres, including waiting rooms; iii) health education sessions by health facility staff for attendees of health facilities
  - Case detection, assessment, and treatment initiation by health centre nurses and health officers, including training of patients in self-care, patient counselling and coping skills acquisition, and mental health care
  - Clinical mentoring for facility-based health workers
  - Supportive supervision for facility-based health workers and CHWs
- Community:
  - Capacity-building for CHWs: i) training on lymphoedema MMDP and mental health care provision; ii) Community Conversation (CC) facilitator training
  - Community awareness-raising: i) awareness-raising workshops for members of the community by CHWs; ii) Community Conversation groups; iii) information dissemination in the community by CHWs (information leaflets, posters in health posts, mass media)
  - Active case detection and referral by CHWs
  - Patient follow-up visits by CHWs
  - Community-based rehabilitation, including home visits, meetings, workshops, patient associations and self-help groups, family support groups, and community mobilisation
  - Supportive supervision for CHWs

In addition, cutting across the three levels of the health care system, there may also be a community advisory group of a wide range of stakeholders across the three levels. It will be important to ensure high quality of the health care providers so that they are able to combine physical health, mental health, and psychosocial care.

Piloting of care package during Phase 2

During the pilot study in one sub-district in Awi zone, the main training elements and interventions of the care package are tested, to establish their adoption, feasibility, acceptability, fidelity, potential effectiveness, their readiness for scale up, costs of the interventions, and the suitability of the training and training materials. This involves testing the interventions, including their training components, as outlined in the section on the draft care package above.

During the pilot study, the interventions covered by the training are implemented for three months, with evaluations at baseline, one-month and three-month follow-up. See Table 1 for the outcomes measured.

For peer review only - http://bmjopen.bmj.com/site/about/guidelines.xhtml

The care package, as well as the training materials, may be revised further based on the results of the pilot study (success criteria will be established a priori), and there may be further pilot-testing if major challenges are encountered that will need to be addressed further, before being scaled up in three whole districts.

Scale-up of care package during Phase 3

The scale-up and evaluation of the care package during Phase 3 of the EnDPoINT study will involve implementation of all interventions at all three levels of the health system (health care organisation/coordination, health facility and community level) in three districts in Awi zone. Whilst the exact interventions that will be included in the care package for scale-up will depend on the findings of Phases 1 and 2, the general potential structure of the training for the various interventions included in the care package is depicted in Figure 3.

Figure 3 around here

The scale-up of the care package interventions during Phase 3 will be implemented, followed up and evaluated over a period of one year. Patients will be followed up every month for the first three months and then at 12 months. Data will be collected for evaluation at baseline, and at 3- and 12-month follow-up, in regards to coverage, as well as implementation, clinical (physical health, mental health and psychosocial), economic (including total costs of the care package) and social outcomes (see Table 1). A detailed monitoring and evaluation plan for Phase 3 will be developed based on the results in Phase 2.

Outcomes to be measured

Table 1 details the outcomes measured during each of the three phases of EnDPoINT, as well as the time points and methods that will be used to measure these.

Data analysis plan

Qualitative data (such as from the key informant interviews and focus group discussions) will be analysed using thematic analysis with the assistance of a qualitative software package (NVivo). Validity (dependability) of data will be assessed through the identification of the initial codes by an external experienced qualitative researcher and agreeing on the framework in which the data will be presented, presentation of initial findings to experienced academics to assess the plausibility, and including single counting of identified events/phenomena. Validity assurance will also involve checking of alignment of research questions, interview guides, sampling procedures etc. For quantitative data collected during the Phase 2 pilot study, descriptive analyses (such as simple counts and frequencies or means and standard deviations) will be used, as well as the % change before and after the implementation of the interventions, together with 95% confidence intervals. A detailed data analysis plan will be produced for the evaluation of the care package during Phases 2 and 3. The results from the various research activities throughout the study will be triangulated to

For peer review only - http://bmjopen.bmj.com/site/about/guidelines.xhtml

develop and finalise the holistic care package and strategies for its integration into the routine health care delivery system in Awi zone in Ethiopia.

Patient and public involvement

The EnDPoINT study is being supported by a Consortium, whose remit is to provide input throughout the programme of research, and which includes patient representatives. Affected people and their families are included in all aspects of the study, including within the workshops and qualitative aspects of the work. Furthermore, patients are partnering with the EnDPoINT study team for the design of the study and care package, and the training materials to support the development of the care package. The EnDPoINT Consortium will contribute to various outputs for dissemination. Patients will therefore be involved in the entire study process, both as co-researchers and as participants of the study.

Economic analysis

We aim to cost the holistic package of physical health, mental health and psychosocial care interventions for people with lymphoedema, and the integration of this package into the routine health care delivery system (Phases 2 and 3), thereby ensuring that the care package represents value for money. Intervention activities will be costed at three levels:

1. Health care organisation/coordination level: awareness-raising activities, programme management and training health care organisation staff;
2. Health care facility level: awareness-raising activities, case detection, treatment, staff training in health education for patient physical health self-care, mental health and psychosocial support;
3. Community level: awareness-raising activities, training and supervision of CHWs, active case detection by CHWs, providing community-based health care and rehabilitation.

The main cost components will be identified in the pilot study (Phase 2); cost data will be collected during the scale-up of the care package intervention (Phase 3). A micro-costing approach will include a bottom-up construction of costs associated with setting-up and delivering the package. The costs of training healthcare professionals will include: time spent in preparing and attending the training sessions, room rental, training materials, travel expenses, and administration costs. The costs of delivering the healthcare package will include: costs of lymphoedema treatment supplies (e.g. soap, ointment, bandages, gloves, antiseptics), infrastructure costs, and additional staff salaries/time. Costs will be collected from financial records and interviews with programme managers.

For peer review only - http://bmjopen.bmj.com/site/about/guidelines.xhtml

ETHICS AND DISSEMINATION

Ethics

Ethical approval has already been obtained from the Brighton and Sussex Medical School Research Governance and Ethics Committee (BSMS RGEC) in the UK, as well as the Institutional Review Board of the College of Health Sciences at Addis Ababa University in Ethiopia.

All people who are participating in the evaluation of the research (all over 18 years old) are given a participant information sheet (outlining the study's research aims, procedures, and potential risks and benefits) and are required to give their written consent to take part (or verbal consent with a witness declaration by the interviewer where a person is illiterate); if a person does not consent to take part, that person is excluded. However, those people who are involved purely in the implementation of the care package, for example those who are receiving training on self-care for lymphoedema or who are taking part in a stakeholder meeting, are not required to give their informed consent, though they are not obliged to be involved. All participants are able to withdraw from the study at any time without having to give a reason or any detrimental consequences, for instance in regard to their care, treatment or employment, until data have been aggregated.

Confidentiality and anonymity of data is ensured throughout the study process, from data collection to data storage, analysis and publication. Structured, multi-level precautions are taken to safeguard the confidential nature of the information gathered, and to ensure the anonymity of participants, for example by using personal identifiers instead of names, with identifying data kept separately from the identifying codes used on the data collection sheets and databases. Only anonymised data will be used for data analyses.

Dissemination

An EnDPoINT publication plan has already been developed, which includes several planned articles for publication in scientific journals. It is likely that the results of the study will also be shared through various other forums, including to the larger scientific community through conferences, but also to policymakers and health care staff, and other participants of the study. This will likely be in the form of a workshop at the end of the study and/or through the distribution of a summary document and/or policy brief(s) with an overview of the main results of the study.

For peer review only

For peer review only - http://bmjopen.bmj.com/site/about/guidelines.xhtml

References

1. Deribe K, Cano J, Giorgi E, et al.; Estimating the number of cases of podoconiosis in Ethiopia using geostatistical methods. *Wellcome Open Research* 2017;**2**:78.
2. International Federation of Anti-Leprosy Associations (ILEP). https://www.ilepfederation.org/ (Date Accessed 2020 Accessed, date last accessed)
3. Rebollo M, Sime H, Assefa A, et al.; Shrinking the Lymphatic Filariasis Map of Ethiopia: Reassessing the Population at Risk through Nationwide Mapping. *PloS NTDs* 2015;**9**(11):e0004172. doi: 10.1371/journal.pntd.000417.
4. Sime H, Gass K, Mekasha S, et al.; Results of a confirmatory mapping tool for Lymphatic filariasis endemicity classification in areas where transmission was uncertain in Ethiopia. *PloS NTDs* 2018;**12**(3):e0006325.
5. Kebede B, Martindale S, Mengistu B, et al.; Integrated morbidity mapping of lymphatic filariasis and podoconiosis cases in 20 co-endemic districts of Ethiopia. *PloS NTDs* 2018;**12**(7):e0006491. doi: doi: 10.1371/journal.pntd.0006491.
6. Deribe K, Brooker S, Pullan R, et al.; Epidemiology and Individual, Household and Geographical Risk Factors of Podoconiosis in Ethiopia: Results from the First Nationwide Mapping. *Am. J. Trop. Med. Hyg* 2015;**92**(1):148-158.
7. Ottesen E, Hooper P, Bradley M, et al.; The Global Programme to Eliminate Lymphatic Filariasis: Health Impact after 8 Years. *Plos NTDs* 2008;**2**(10):e317. doi: https://doi.org/10.1371/journal.pntd.0000317.
8. World Health Organization (WHO); Weekly epidemiological record. In: WHOs (ed). Geneva: WHO, 2013, 365-380.
9. Centers for Disease Control and Prevention (CDC). *World Leprosy Day: Bust the Myths, Learn the Facts*. https://www.cdc.gov/features/world-leprosy-day/index.html (Date Accessed 2018 Accessed, date last accessed)
10. Tekola A, Adeyemo A, Finan C, et al.; HLA class II locus and susceptibility to podoconiosis. *New England Journal of Medicine* 2012;**366**:1200-1208.
11. Bekri W, Gebre S, Mengiste A, et al.; Delay in presentation and start of treatment in leprosy patients: a case-control study of disabled and non-disabled patients in three different settings in Ethiopia. *International Journal of Leprosy* 1998;**66**(1):1-9.
12. Mousley E, Deribe K, Tamiru A, et al.; Mental distress and podoconiosis in Northern Ethiopia: a comparative cross-sectional study. *International Health* 2014. doi: 10.1093/inthealth/ihu043.
13. Bartlett J, Deribe K, Tamiru A, et al.; Depression and disability in people with podoconiosis: a comparative cross-sectional study in rural Northern Ethiopia. *International Health* 2016. doi: doi:10.1093/inthealth/ihv037.
14. Semrau M, Davey G, Beng A, et al.; Depressive symptoms amongst people with podoconiosis and lower limb lymphoedema of other cause in Cameroon: A cross-sectional study. *Tropical Medicine and Infectious Disease.* 2019;**4**(3):E102. doi: doi:10.3390/tropicalmed4030102.
15. Hofstraat K, van Brakel W; Social stigma towards neglected tropical diseases: a systematic review. *International Health* 2016;**8**(s1):i53-i70.
16. Tora A, Davey G, Tadele G; A Qualitative Study on Stigma and Coping Strategies of Patients with Podoconiosis in Wolaita Zone, Southern Ethiopia. *International Health* 2011;**3**(3):176-181.
17. Tora A, Franklin H, Deribe K, et al.; Extent of podoconiosis-related stigma in Wolaita Zone, Southern Ethiopia: a cross-sectional study. *Springer Plus* 2014;**3**:647.
18. Person B, Bartholomew L, Gyapong M, et al.; Health-related stigma among women with lymphatic filariasis from the Dominican Republic and Ghana *Social Science & Medicine* 2009;**68**(1):30-38.
19. Tekola F, HaileMariam D, Davey G; Economic costs of endemic non-filarial elephantiasis in Wolaita Zone, Ethiopia. *Tropical Medicine & International Health* 2006;**11**(7):1136-1144.
20. Perera M, Whitehead M, Molyneux D, et al.; Neglected Patients with a Neglected Disease? A Qualitative Study of Lymphatic Filariasis. *PLoS Neglected Tropical Diseases* 2007;**1**(2):128.

For peer review only - http://bmjopen.bmj.com/site/about/guidelines.xhtml

21. Martindale S, Mkwanda S, Smith E, et al.; Quantifying the physical and socio-economic burden of filarial lymphoedema in Chikwawa District, Malawi. *Transactions of the Royal Society of Tropical Medicine & Hygiene* 2014;**108**(12):659-667.
22. Slanton M, Yamauchi M, Mkwanda S, et al.; Measuring the physical and economic impact of filarial lymphoedema in Chikwawa district, Malawi: a case-control study. *Infect Dis Poverty* 2017;**6**(1):28. doi: 10.1186/s40249-017-0241-2.
23. Deribe K, Negussu N, Newport M, et al.; The health and economic burden of podoconiosis in Ethiopia. *Trans R Soc Trop Med Hyg* in press.
24. Hotez P, Fenwick A, Savioli L, et al.; Rescuing the “bottom billion” through neglected tropical disease control. *Lancet* 2009;**373**:1570-1576.
25. Ton T, Mackenzie C, Molyneux D; The burden of mental health in LF. *Infect Dis Poverty* 2015;**4**:34.
26. World Health Organization (WHO). *Lymphatic filariasis - Policy*. https://www.who.int/lymphatic_filariasis/policy/en/ (Date Accessed 2020 Accessed, date last accessed)
27. World Health Organization (WHO). *Leprosy elimination - Elimination of leprosy FAQ*. https://www.who.int/lep/strategy/faqs/en/ (Date Accessed 2020 Accessed, date last accessed)
28. Deribe K, Meribo K, Gebre T, et al.; The burden of neglected tropical diseases in Ethiopia, and opportunities for integrated control and elimination. *Parasites and Vectors* 2012;**5**:240.
29. Obindo J, Abdulmalik J, Nwefoh E, et al.; Prevalence of depression and associated clinical and socio-demographic factors in people living with lymphatic filariasis in Plateau State, Nigeria. *PLOS Neglected Tropical Diseases* 2017;**11**(6):e0005567.
30. Demyttenaere K, Bruffaerts R, Posada-Villa J, et al.; Prevalence, severity, and unmet need for treatment of mental disorders in the World Health Organization World Mental Health Surveys. *JAMA* 2004;**291**:2581–90.
31. Deribe K, Wanji S, Shafi O, et al.; The feasibility of eliminating podoconiosis. *Bull World Health Organ* 2015;**93**(10):712-8.
32. Ministry of Health FDRoE; National Masterplan for Neglected Tropical Diseases (NTDs). Addis Ababa: Ministry of Health FDRoE, 2013, 1-56.
33. World Health Organization (WHO). *Sixty ninth World Health Assembly adopts resolution on mycetoma*. http://www.who.int/neglected_diseases/news/WHA_69_adopts_mycetoma_resolution/en/ (Date Accessed 2016 Accessed, date last accessed)
34. Ayode D, McBride C, de Heer H, et al.; The association of beliefs about heredity with preventive and interpersonal behaviors in communities affected by podoconiosis in rural Ethiopia. *Am J Trop Med Hyg* 2012;**87**(4):623-30.
35. McBride C, Price C, Ayode D, et al.; A cluster randomised intervention trial to promote shoe use by children at high risk for podoconiosis. *Int J Health Sci Res* 2015;**5**(6):518-528.
36. Peters D, Tran N, Adam T; Implementation research in health - A practical guide. Geneva: World Health Organization, 2013.
37. Craig P, Dieppe P, Macintyre S, et al.; Developing and evaluating complex interventions: the new Medical Research Council guidance. *British Medical Journal* 2008;**337**:a1655. doi: 10.1136/bmj.a1655.
38. Medical Research Council (MRC). *Developing and evaluation complex interventions: new guidance*. https://www.mrc.ac.uk/documents/pdf/complex-interventions-guidance/ (Date Accessed, date last accessed)
39. De Silva M, Breuer E, Lee L, et al.; Theory of Change: a theory-driven approach to enhance the Medical Research Council's framework for complex interventions. *Trials* 2014;**15**:267.
40. Breuer E, de Silva M, Fekadu A, et al.; Using workshops to develop theories of change in five low and middle income countries: lessons from the programme for improving mental health car (PRIME). *International Journal of Mental Health Systems* 2014;**8**:15.

For peer review only - http://bmjopen.bmj.com/site/about/guidelines.xhtml

41. Craig P, Dieppe P, Macintyre S, et al.; Developing and evaluating complex interventions: the new Medical Research Council guidance. *BMJ* 2008;**337**:a1655.
42. Office of the Population Census Commission (OPCC); The 2007 Population and Housing Census of Ethiopia. 2007.

For peer review only

For peer review only - http://bmjopen.bmj.com/site/about/guidelines.xhtml

Authors' contributions

All authors contributed to the manuscript and have accepted the final version. MS led the writing of the manuscript. GD and AF are the Principal Investigators of the EnDPoINT study. OAA, AM, AT, MK, SAB and NH are part of the core EnDPoINT research team; OAA, AM, AT and MK are responsible for the implementation of the study in Ethiopia; SAB leads the statistical elements of the study; and NH leads the economic aspects. KD produced two of the figures in the manuscript, and is a member of the EnDPoINT Research Consortium, as are LAK-H, HM, HBT, HB, DH and NN. All members of the EnDPoINT Research Consortium have actively contributed to the inception of the study and continuous deliberations since, in relation to conceptual and methodological approaches and implementation.

Acknowledgements

We thank the other members of the EnDPoINT Research Consortium for their input into the study, as follows: Ms Tsige Amberbir, Ms Tanny Hagens, Mr Mossie Tamiru, Mr Tadesse Tesfaye, Dr Seifu Tirfie, and Dr Abebayehu Tora. We also thank Ms Clare Callow for her project management of the NIHR grant through which EnDPoINT is funded, as well as Mr Tesfaye Asefa, Ms Bethelhem Fekadu, Ms Grit Gansch, Ms Samrawit Ketema for their support with the administrative aspects of the study. Our sincerest thanks to all participants of the EnDPoINT study.

Funding

This research was commissioned by the National Institute for Health Research (NIHR) Global Health Research Unit on NTDs at BSMS using Official Development Assistance (ODA) funding. The views expressed in this publication are those of the author(s) and not necessarily those of the NIHR or the Department of Health and Social Care. KD is supported by the Wellcome Trust as part of his International Intermediate Fellowship [grant number 201900].

Competing interests

The authors have no competing interests to declare.

For peer review only

Table 1 Objectives, research questions, research activities and outcome measures for each of the three phases within the EnDPoINT study

	Objectives	Research questions	Research activities	Outcome measures
**Phase 1 – Development of care package**	• Finalise a comprehensive package of holistic physical health, mental health and psychosocial care for people with podoconiosis, LF and leprosy<br>• Learn lessons about integration from implementers and beneficiaries of care to date<br>• Develop strategies for integrating and evaluating the holistic care package into the routine health care delivery system in selected districts in Awi zone in Ethiopia, and integrating care across the three diseases	• What are the key elements that constitute optimal physical health, mental health and psychosocial care for patients with podoconiosis, LF and leprosy?<br>• What strategies need to be developed to facilitate integration of the holistic care package into the routine health care delivery system and across the three diseases?<br>• Is the care package feasible, acceptable and appropriate in terms of its integration into government-run health services?	• *Document review* of grey literature, including existing national NTD guidelines, other relevant documents on care provision for NTDs and/or mental health, study reports and programme documents, to inform and guide the development of the care package.<br>• *Systematic review* of publications in scientific journals, on the functional/disability, mental health and psychosocial outcomes secondary to podoconiosis, LF and leprosy, to complement the document review.<br>• *Situational analysis / resource mapping*, to collect cross-sectional baseline data on contextual factors relevant to the development, implementation and integration of the care package, as well as to identify any resources available for this, and potential risk factors.<br>• *Three 'Theory of Change' (ToC) workshops* with members of the research team and key stakeholders, to identify and establish the key causal pathways between the desired outcomes, interventions, assumptions, indicators and measurement of the outcomes for the care package (represented visually through a ToC map), as well as to encourage stakeholder buy-in to the study (39).<br>• *Key informant interviews and focus group discussions* with stakeholders, to assess the draft care package's feasibility (i.e. the extent to which the intervention can be carried out within the	Qualitatively assessed implementation outcomes, including feasibility, acceptability and appropriateness.

For peer review only - http://bmjopen.bmj.com/site/about/guidelines.xhtml

			routine health system), acceptability (i.e. the perception among stakeholders that the care package is agreeable), and appropriateness (i.e. the perceived fit or relevance of the care package to key stakeholders), and to assess key aspects of the ToC (for example, assumptions made).<br>• *Workshop* with key stakeholders to discuss the draft *care plan and training materials* that will be adapted.<br>• Informed by the above steps, *develop a comprehensive care package* consisting of interventions at the healthcare organisation/coordination, health facility and community level.	
**Phase 2 – Piloting of care package in one sub-district**	• Implement and evaluate the care package in one sub-district in Awi zone in Ethiopia<br>• Develop a monitoring and evaluation plan for the subsequent scale-up of implementation of the care package	• What are the critical contextual factors (including drivers and barriers) that influence the process of integration of the holistic care package into government-run health services and across the three diseases?<br>• What are the key features of the intervention that influence the manner of integration into the health care system and across the three diseases?<br>• Who are the key health system actors that have a stake in the integration of the care package into the government-run health	• *Pilot study of the care package in one sub-district* in Awi zone, to assess its adoption (i.e. the intention of trying to employ the care package), feasibility, acceptability, fidelity (i.e. the degree to which the care package was implemented as designed), effectiveness (i.e. the impact of the care package as delivered on individual patient outcomes), costing of the care package, its readiness for scale-up, and the suitability of the training and training materials. This is achieved through:<br>○ Observation<br>○ Key informant interviews and/or focus group discussions with key stakeholders such as people who received the training and/or those delivered or received the	• Qualitatively assessed implementation outcomes, including adoption, feasibility, acceptability and fidelity.<br>• Effectiveness of care package interventions assessed at baseline, one-month and 3-month follow-up, indicated by number of cases identified and treated, and patient-level outcomes (pre-post) through structured questionnaires, including MMDP assessment, swelling circumference, frequency of acute attacks, stage and grade of affected

For peer review only

		services, and what coordination and capacity building needs exist?<br>• Is the holistic care package adoptable, feasible, effective, and of high fidelity when integrated into government-run health services and across the three diseases?<br>• What are the observable trends in the utilisation and coverage of the care package?	interventions; cost data will be collected from programme managers.<br>○ Before-and-after (pre-post) collection of quantitative data, including number of cases identified and treated (and whether there were any differences in the way these were identified), patient-level outcomes, and for the training 'change of knowledge, attitudes and practice' (KAP) questionnaires<br>○ Evaluation of the delivery of the training<br>• *Workshop* with the NTD Department of the Ethiopian FMOH<br>• Based on the pilot study and workshop, *development of a protocol to evaluate scale-up* of integration of the holistic care package across the three diseases into the state health system in three districts in Awi zone in Ethiopia.	limb, depression (measured through the Patient Health Questionnaire, PHQ-9), suicidal ideation (CIDI), alcohol use (FAST), quality of life (Dermatology Quality of Life Index, DQLI), disability (WHODAS 2.0), internalised stigma (ISRL), discrimination (DISC-12), social distance (Social Distance Scale, SDS), social support (OSLO 3), happiness index, and explanatory models.<br>• Training evaluations, measured by questionnaires on 'change of knowledge, attitudes and practice' (KAP) (immediately before and after training), and mixed-methods satisfaction data (immediately after training).<br>• Cost of interventions (economic outcomes).
**Phase 3 – Scale-up and evaluation of care package in three districts**	• Scale up the holistic care package across the three diseases into government-run health services in three districts in Awi zone in Ethiopia based on the findings from the pilot	• What are the critical factors (including drivers and barriers) that influence the process of scaling-up the care package, and that ensure its effectiveness, sustainability, quality and coverage?	• Implementation of the interventions that are included in the care package and that were developed and piloted during Phases 1 and 2 of the study. These will include interventions at the health care organisation/coordination, health facility and community levels.<br>• Evaluation of care package.	The exact evaluation plan will be developed and finalised following Phases 1 and 2, but will include evaluation of the care package in regards to coverage (i.e. the degree to which patients in the selected districts actually received the care package), as well as

For peer review only

	study in the single sub-district (during Phase 2)<br>• Evaluate the scale-up of the care package<br>• Conduct analysis of the intervention costs during the scale-up	• How does the context interact with the intervention to influence the effectiveness of integration, i.e. how do these elements fare in the different contexts presented by the three districts?<br>• Does the care package result in improved outcomes for patients with podoconiosis, LF and leprosy, including clinical (physical health, mental health and psychosocial), economic and social outcomes?<br>• What is the economic impact of the care package?		implementation, clinical (physical health, mental health and psychosocial), economic and social outcomes, similar to the evaluation during the pilot study (see above). Evaluation data will be collected at baseline, and at 3- and 12-month follow-up.

For peer review only - http://bmjopen.bmj.com/site/about/guidelines.xhtml

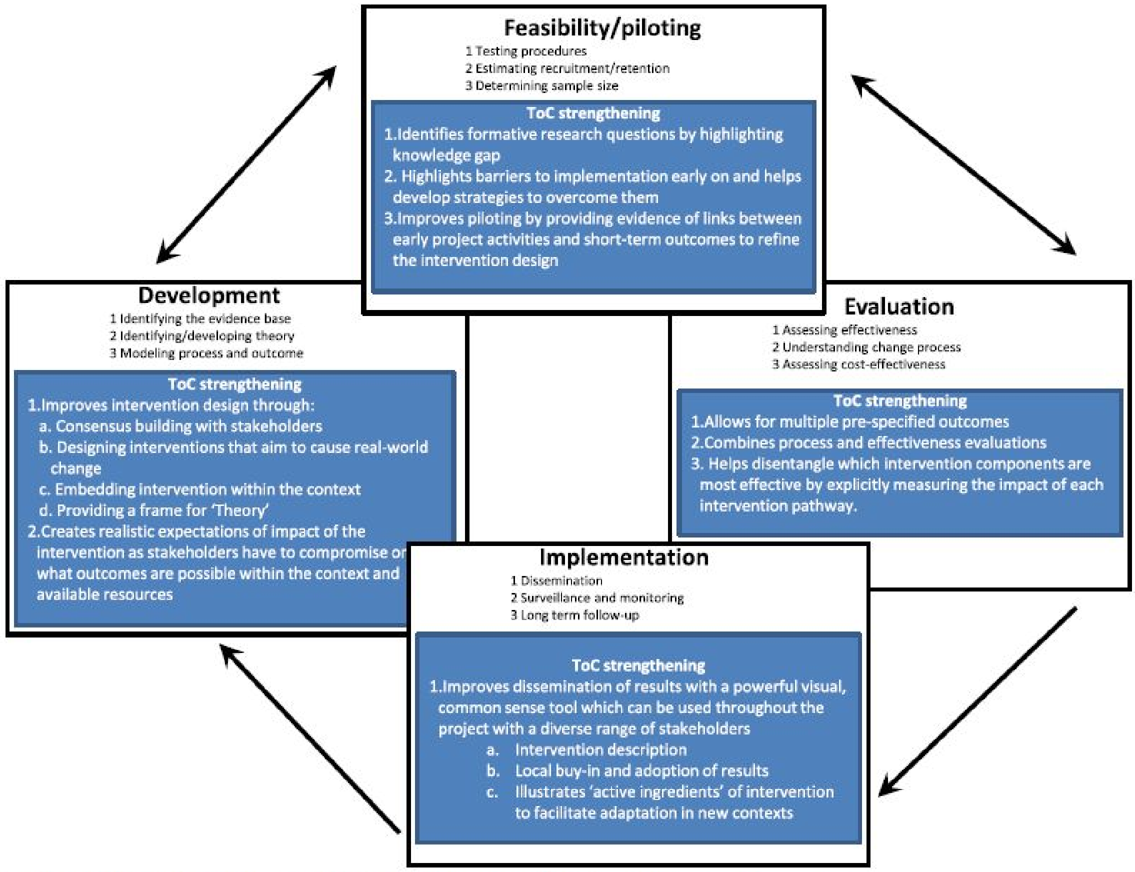

Figure 2 How Theory of Change can be used to strengthen the MRC framework. Adapted from Craig *et al.* [1].

Figure 1: 'Theory of Change' within the MRC framework on complex interventions (taken from de Silva et al 2014 (39)) (in white: taken directly from MRC framework; in blue: added elements on ToC)

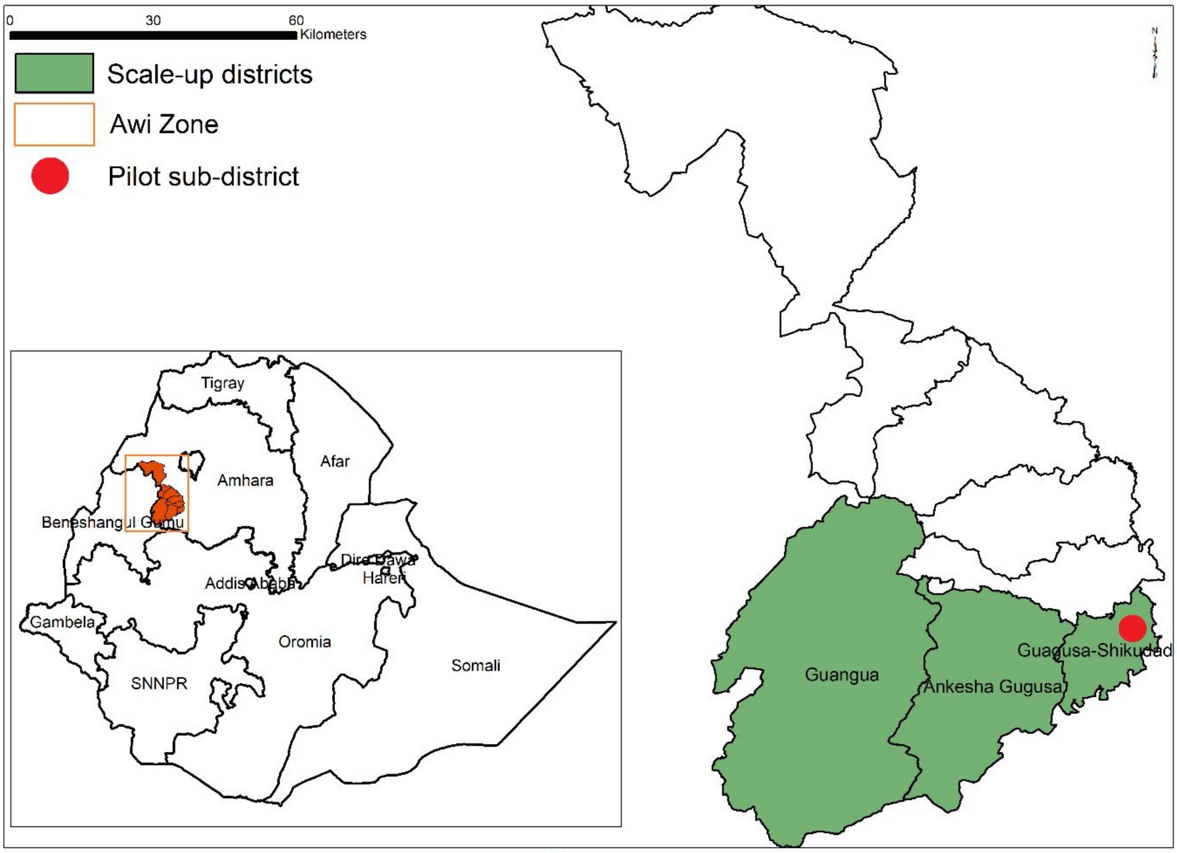

Figure 2: Implementation districts for the EnDPoINT study

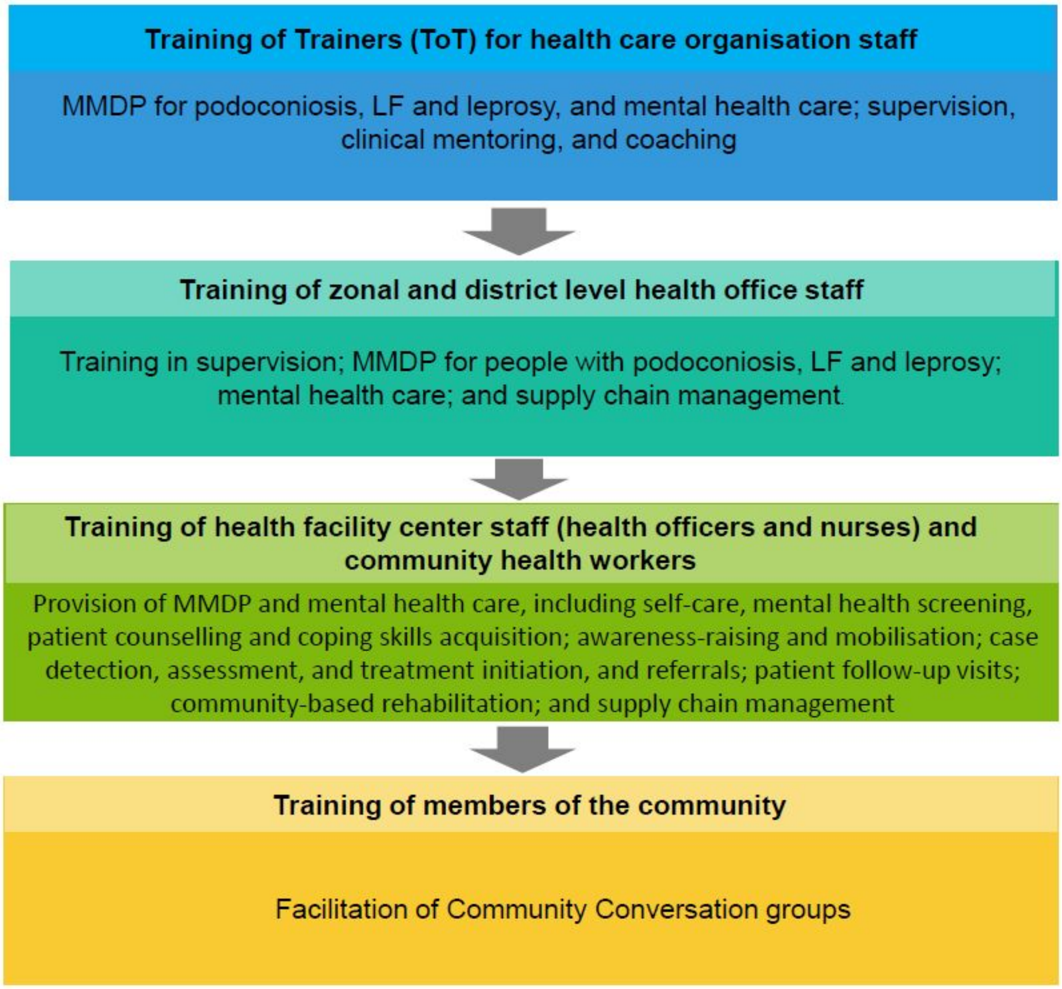

Figure 3: Potential training structure within the EnDPoINT study

BMJ Open

EnDPoINT – Protocol for an implementation research study to integrate a holistic package of physical health, mental health and psychosocial care for podoconiosis, lymphatic filariasis and leprosy into routine health services in Ethiopia

Journal:	*BMJ Open*
Manuscript ID	bmjopen-2020-037675.R1
Article Type:	Protocol
Date Submitted by the Author:	14-Aug-2020
Complete List of Authors:	Semrau, Maya; Brighton and Sussex Medical School, Centre for Global Health Research<br>Ahmed, Oumer; Brighton and Sussex Medical School, Centre for Global Health Research; Addis Ababa University, Center for Innovative Drug Development and Therapeutic Trials for Africa (CDT-Africa)<br>Deribe, Kebede; Brighton and Sussex Medical School, Centre for Global Health Research; Addis Ababa University, School of Public Health, College of Health Sciences<br>Mengiste, Asrat; Addis Ababa University, Center for Innovative Drug Development and Therapeutic Trials for Africa (CDT-Africa)<br>Tesfaye, Abraham; Addis Ababa University, Center for Innovative Drug Development and Therapeutic Trials for Africa (CDT-Africa)<br>Kinfe, Mersha; Addis Ababa University, Center for Innovative Drug Development and Therapeutic Trials for Africa (CDT-Africa)<br>Bremner, Stephen; Brighton and Sussex Medical School, Department of Primary Care and Public Health<br>Hounsome, Natalia; Brighton and Sussex Medical School, Centre for Global Health Research<br>Kelly-Hope, Louise; Liverpool School of Tropical Medicine, Centre for Neglected Tropical Diseases, Department of Tropical Disease Biology<br>MacGregor, Hayley ; Institute of Development Studies<br>Taddese, Henock; Imperial College London, Faculty of Medicine, School of Public Health<br>Banteyerga, Hailom ; Addis Ababa University, College of Humanities, Language Studies, Journalism and Communication<br>HaileMariam, Damen; Addis Ababa University, School of Public Health, College of Health Sciences<br>Negussu, Nebiyu; Federal Ministry of Health<br>Fekadu, Abebaw; Brighton and Sussex Medical School, Centre for Global Health Research; Addis Ababa University, Center for Innovative Drug Development and Therapeutic Trials for Africa (CDT-Africa)<br>Davey, Gail; Brighton and Sussex Medical School, Centre for Global Health Research
**Primary Subject Heading**:	Global health
Secondary Subject Heading:	Health services research, Infectious diseases, Mental health

Keywords:	MENTAL HEALTH, INFECTIOUS DISEASES, PRIMARY CARE, PUBLIC HEALTH

SCHOLARONE™
Manuscripts

I, the Submitting Author has the right to grant and does grant on behalf of all authors of the Work (as defined in the below author licence), an exclusive licence and/or a non-exclusive licence for contributions from authors who are: i) UK Crown employees; ii) where BMJ has agreed a CC-BY licence shall apply, and/or iii) in accordance with the terms applicable for US Federal Government officers or employees acting as part of their official duties; on a worldwide, perpetual, irrevocable, royalty-free basis to BMJ Publishing Group Ltd ("BMJ") its licensees and where the relevant Journal is co-owned by BMJ to the co-owners of the Journal, to publish the Work in this journal and any other BMJ products and to exploit all rights, as set out in our licence.

The Submitting Author accepts and understands that any supply made under these terms is made by BMJ to the Submitting Author unless you are acting as an employee on behalf of your employer or a postgraduate student of an affiliated institution which is paying any applicable article publishing charge ("APC") for Open Access articles. Where the Submitting Author wishes to make the Work available on an Open Access basis (and intends to pay the relevant APC), the terms of reuse of such Open Access shall be governed by a Creative Commons licence – details of these licences and which Creative Commons licence will apply to this Work are set out in our licence referred to above.

Other than as permitted in any relevant BMJ Author's Self Archiving Policies, I confirm this Work has not been accepted for publication elsewhere, is not being considered for publication elsewhere and does not duplicate material already published. I confirm all authors consent to publication of this Work and authorise the granting of this licence.

For peer review only

EnDPoINT –
Protocol for an implementation research study to integrate a holistic package of physical health, mental health and psychosocial care for podoconiosis, lymphatic filariasis and leprosy into routine health services in Ethiopia

Semrau M[1], Ali O[1,2], Deribe K[1,3], Mengiste A[2], Tesfaye A[2], Kinfe M[2], Bremner SA[4], Hounsome N[1], Kelly-Hope LA[5], MacGregor H[6], Taddese HB[7], Banteyerga H[8], HaileMariam D[3], Negussu N[9], Fekadu A[1,2*], Davey G[1*]

[1] Centre for Global Health Research, Brighton and Sussex Medical School, Brighton, United Kingdom

[2] Center for Innovative Drug Development and Therapeutic Trials for Africa (CDT-Africa), Addis Ababa University, Addis Ababa, Ethiopia

[3] School of Public Health, College of Health Sciences, Addis Ababa University, Addis Ababa, Ethiopia

[4] Department of Primary Care and Public Health, Brighton and Sussex Medical School, Brighton, United Kingdom

[5] Centre for Neglected Tropical Diseases, Department of Tropical Disease Biology, Liverpool School of Tropical Medicine, Liverpool, United Kingdom

[6] Institute of Development Studies, Brighton, United Kingdom

[7] Faculty of Medicine, School of Public Health, Imperial College London, London, United Kingdom

[8] College of Humanities, Language Studies, Journalism and Communication; Addis Ababa University, Addis Ababa, Ethiopia

[9] Federal Ministry of Health, Addis Ababa, Ethiopia

[*] joint last authors

Corresponding author: Dr Maya Semrau, Global Health and Infection Department, Room G19, Medical Research Building, Brighton and Sussex Medical School, University of Sussex, Falmer, Brighton, BN1 9PS, United Kingdom; email: m.semrau@bsms.ac.uk; phone: +44-1273-872788

Word count: 5769

For peer review only - http://bmjopen.bmj.com/site/about/guidelines.xhtml

ABSTRACT

Introduction: Neglected tropical diseases (NTDs) causing lower-limb lymphoedema such as podoconiosis, lymphatic filariasis (LF) and leprosy are common in Ethiopia. Routine health services for morbidity management and disability prevention (MMDP) of lymphoedema caused by these conditions are still lacking, even though it imposes a huge burden on affected individuals and their communities in terms of physical and mental health, and psychosocial and economic outcomes. This calls for an integrated, holistic approach to MMDP across these three diseases.

Methods and analysis: The 'Excellence in Disability Prevention Integrated across NTDs' (EnDPoINT) implementation research study aims to assess the integration and scale-up of a holistic package of care – including physical health, mental health, and psychosocial care – into routine health services for patients with lymphoedema caused by podoconiosis, LF and leprosy in selected districts in Awi zone in the North-West of Ethiopia. The study is being carried out over three phases using a wide range of mixed methodologies. Phase 1 involves the development of a comprehensive holistic care package and strategies for its integration into the routine health services across the three diseases, and to examine the factors that influence integration and the roles of key health system actors. Phase 2 involves a pilot study conducted in one sub-district in Awi zone, to establish the care package's adoption, feasibility, acceptability, fidelity, potential effectiveness, its readiness for scale-up, costs of the interventions, and the suitability of the training and training materials. Phase 3 involves scale-up of the care package in three whole districts, as well as its evaluation in regard to coverage, implementation, clinical (physical health, mental health and psychosocial), and economic outcomes.

Ethics and dissemination: Ethics approval for the study has been obtained in the UK and Ethiopia. The results will be disseminated through publications in scientific journals, conference presentations, policy briefs and workshops.

Key words: lymphoedema, podoconiosis, lymphatic filariasis, leprosy, mental health, implementation research

For peer review only - http://bmjopen.bmj.com/site/about/guidelines.xhtml

ARTICLE SUMMARY

Strengths and limitations of this study

- The EnDPoINT implementation research study takes a holistic approach in that it explores the full scope of processes and outcomes involved in the integration of physical health, mental health, and psychosocial care for people with lower-limb lymphoedema caused by podoconiosis, lymphatic filariasis (LF) and leprosy; integration of care relates to: i) integration of care across the three diseases; ii) integration of care for these diseases into routine health services; and iii) integration of mental health and psychosocial (including stigma reduction) interventions into holistic packages of care.
- EnDPoINT is guided by relevant conceptual perspectives on implementation research as well as employing a 'Theory of Change' (ToC) approach towards devising, managing and evaluating the integration study.
- The study uses a wide range of mixed-methods approaches across three phases to develop, scale-up and evaluate the holistic care package.
- Limitations of the study are: selection of study districts partly based on accessibility; time and resource constraints that do not allow the pilot study to have a control group or full-length follow-up before the scale-up; and possible contamination of the study results by previous or ongoing work of other organisations in the study zone.
- The study team comprises an array of multi-disciplinary academics and professionals in Ethiopia as well as internationally, including medics, epidemiologists, psychiatrists, policy experts, social scientists, participatory intervention methodologists, and patient representatives.

For peer review only

For peer review only - http://bmjopen.bmj.com/site/about/guidelines.xhtml

INTRODUCTION

Neglected tropical diseases (NTDs) that result in lower limb lymphoedema, that is, swelling of the lower leg, include podoconiosis, lymphatic filariasis (LF) and leprosy. These conditions are common in Ethiopia, with an estimated 1.53 million cases of podoconiosis in the country (1), and around 300,000 people affected by leprosy (2). The exact prevalence for LF lymphoedema in Ethiopia is less well known, since recent mapping studies have resulted in differing estimates (3, 4); however integrated morbidity mapping in 2018 in 20 podoconiosis-LF co-endemic districts identified a prevalence of 84.9 per 10,000 population (26,123 cases in total), of whom 95.3% had leg lymphoedema only, 2.9% had hydrocele (swelling of the genitals in men), 1.5% had both leg lymphoedema and hydrocele, and 0.3% cases had breast lymphoedema (5). Nationwide mapping in 2013 demonstrated that podoconiosis accounts for approximately 64.8%, LF for 13.2%, and leprosy for 12.8% of the total burden of lymphoedema in Ethiopia (6). With global burden estimates of 15 million, 4 million and 2-3 million for LF lymphoedema, podoconiosis and people affected by leprosy respectively (7-9), improving morbidity management and disability prevention (MMDP) and enhancing our understanding of processes and outcomes of these efforts, in particular how to integrate MMDP into existing routine primary community-based health care services, are likely to be of global significance and address resource and sustainability questions (10).

Lymphoedema imposes huge burdens on affected individuals and their communities in terms of disability such as reduced mobility and pain (11), mental distress (12), depression and anxiety (13, 14), stigma, discrimination and social exclusion (11, 15-18), which can limit health-seeking behaviours and access to social services including education, thereby leading to loss of economic productivity at household, community and national level (19-22). For podoconiosis in Ethiopia alone, affected people lose on average 45% of their economically productive time due to morbidity associated with the disease (20), and the estimated cost to Ethiopia's economy is $213 million per year (23). These conditions therefore compromise the livelihoods and wellbeing of populations that are already disadvantaged and hard-to-reach, for example because they live remotely (24). Burden of disease measures are even higher when taking into account the psychological and emotional consequences of NTDs, with estimates that the burden of these psychosocial outcomes may be double that of the physical health consequences (25).

Along with the mental distress and disorder that commonly accompanies these NTDs, stigma is another key issue that significantly increases the disease burden for these diseases (26), and which acts as major barrier to accessing MMDP services (27, 28). It is now widely recognized that joint approaches to reduce stigmatization across NTDs may be feasible given the similarities in causes, manifestations and interventions (29), but there remains a knowledge gap in regards to relevant, evidence-based stigma reduction interventions for use within integrated MMDP programmes. Prior research suggests that stigma for lower limb lymphoedema has three main causes: i) misinformation amongst the community, patients and their families about the diseases' causes, treatment and prevention (30, 31), which could be addressed through educational interventions providing standardized health information, to increase disease-related health literacy; ii) the common poverty and reduced quality of life due to affected individuals' lost economic productivity (32), which could be addressed through community-based socio-economic rehabilitation/strengthening of affected individuals and their families; and iii) the economic burden related to the costs of care, including transport to health facilities (30, 33), which could be lessened by providing integrated services in nearby health facilities at low or no cost. Since multi-component interventions are more effective than

For peer review only - http://bmjopen.bmj.com/site/about/guidelines.xhtml

single-component interventions for stigma reduction, it is important for programmes to address all three causes of stigma (which the project described here attempts to do).

The World Health Organization (WHO) has published targets for elimination of many NTDs, including LF and leprosy (34, 35); however, this has not included podoconiosis (though podoconiosis has been included under LF for MMDP). Whilst foot care for leprosy patients has been integrated into routine health services in Ethiopia since 2001 (36), foot care interventions for podoconiosis and LF aiming to prevent disability are currently mainly provided through donor-supported projects in a disease-specific and disparate manner. Knowledge of these neglected diseases within health care systems is therefore often inadequate, diagnosis and treatment options are limited, and the cost of accessing health care to ease symptoms can be prohibitively expensive. On the other hand, the Ethiopian Federal Ministry of Health (FMOH) has developed integrated guidelines for LF and podoconiosis MMDP but these did not include leprosy (nor did they include mental health or psychosocial components). Yet, there is a clear rationale for the integration of care across these three conditions because of the shared clinical symptomatology of lower-leg lymphoedema. In addition, as the mental distress and illness that commonly accompanies lymphoedema (12, 13, 37) often goes untreated (38), care can be enhanced further for these patients by adding mental health and psychosocial components to physical health services. Integration of care for lymphoedema can therefore include three aspects: i) integration of care across the three diseases; ii) integration of care for these diseases into routine health services; and iii) integration of mental health and psychosocial (including stigma reduction) interventions into holistic packages of care. Integrated care models for NTDs have also been used in other countries (39, 40).

The Ethiopian FMOH has recognized the importance of these issues, and as a result, both podoconiosis and LF have now been included in the first two National Master Plans (2013-2015 and 2015/16-2019/20) for integrated control of NTDs (41, 42) and identified as two of eight priority NTDs in the country, whilst at the same time aiming to move away from vertical programmes. There have also been calls to improve the responsiveness of the health system and include mental health and psychosocial care into the MMDP of podoconiosis and other lymphoedema in Ethiopia (12). The Health Extension Programme, rolled out successfully in Ethiopia since 2003, now boasts more than 38,000 community-based Health Extension Workers (HEWs), and a supervisory system to support them (43). Their reach has been extended through the women-centred Health Development Army (HDA), members of which link 'model families' with five other households to implement health initiatives (33). These cadres are ideally placed to offer simple, low-tech foot care as well as mental health and psychosocial support to people living with lymphoedema in Ethiopia (44), and to refer on patients who need more specialised care.

To address these issues and make use of the opportunities that currently exist in Ethiopia, the FMOH requested implementation research to guide integration of a holistic care package, including physical health, mental health and psychosocial care interventions, for patients with lower-limb lymphoedema into government-run health services. The ‘Excellence in Disability Prevention Integrated across NTDs’ (EnDPoINT) research study (2017-2021), funded by the National Institute for Health Research (NIHR) in the UK, was set up in response to this and our findings are expected to inform existing plans for scale-up of integrated physical limb care and mental health and psychosocial support interventions across Ethiopia. The protocol for the EnDPoINT study is outlined below.

For peer review only - http://bmjopen.bmj.com/site/about/guidelines.xhtml

METHODS AND ANALYSIS

Conceptual framework for study

EnDPoINT is an implementation research study in that its research questions have a strong focus on implementation strategies, it includes a wide range of implementation measures, and it is being conducted in real-world settings and populations (45). The study takes guidance both from the Context and Implementation of Complex Interventions (CICI) framework, as well as from the Medical Research Council (MRC) framework for development and evaluation of complex interventions (46, 47), with further explication and reinforcement of the different steps within the MRC framework through a 'Theory of Change' (ToC) approach (48).

The CICI is a comprehensive framework that incorporates implementation, context and setting as dimensions, and interventions are seen to be operationalised within the macro, meso and micro levels. A core interest of EnDPoINT is to generate transferable learning as to the mechanisms at work during integration of targeted interventions into health systems within low- and middle-income countries. As such, the study is a theory-led investigation, for which the CICI can provide a useful framework. We hope to generate rich descriptions of processes and mechanisms of effect, within the Ethiopian context, thereby enabling inference and transferability to similar settings and conditions.

The MRC framework for complex interventions provides a model for the practical steps involved in the development and evaluation of EnDPoINT's care package (sitting within the broader theoretical framework provided by the CICI). The MRC framework has been used widely and proposes four phases in the development and evaluation of interventions: i) development, ii) feasibility/piloting, iii) evaluation, and iv) implementation; these phases are considered to be an iterative rather than a linear process, and can facilitate locally appropriate strategies to be generated. ToC is a structured thinking process to build a 'causal pathway' of what needs to change and why, in order to achieve the desired outcomes (48); it enhances the MRC framework for complex interventions through its emphasis on a theory-driven approach, which can be incorporated into and provide practical guidance for the different phases of the MRC framework (48). Figure 1 shows how ToC may theoretically link in and strengthen each of the four phases of the MRC framework on complex interventions. Within the EnDPoINT study, the ToC approach will be used within two of the four phases of the MRC framework: the development and feasibility/piloting phases; for these two phases, there is good evidence for the practical viability and usefulness of an embedded ToC approach (49).

Figure 1 around here

Study design

EnDPoINT's aims are to facilitate effective access to physical health, mental health and psychosocial care for patients with lower-limb lymphoedema caused by podoconiosis, LF and leprosy in selected districts in Awi zone in Ethiopia through integration and scale-up of a holistic care package into government-run health services.

The study is organised according to three phases, which map onto the different phases of the MRC framework on complex interventions (47, 50). It takes an iterative approach, whereby the subsequent

For peer review only - http://bmjopen.bmj.com/site/about/guidelines.xhtml

phases are dependent on findings made during the previous phases. See Table 1 for the objectives, research questions, research activities/methods and outcome measures for each of the three phases.

Phase 1 of EnDPoINT (Months 1-20 of the project) – which corresponds to Phase 1 of the MRC framework – entails various research activities to inform the development of the holistic care package. More specifically, the EnDPoINT care package builds on an MMDP (physical) self-care package that has previously been established and tested in Ethiopia for podoconiosis (51), but with the addition of mental health and psychosocial components, as well as enabling provision of integrated care across the three diseases. The development of the care package within Phase 1 thereby relates to: 1) the identification and design of these added mental health and psychosocial elements, whilst 2) integrating care across the three conditions, as well as 3) identifying strategies for integration of the care package into the government-run health care delivery system (as MMDP services are not yet routinely provided in Ethiopia for podoconiosis and LF). A bottom-up approach is used for this, taking into account a wide range of stakeholder views within multiple fora (see Table 1).

Phase 2 (Months 21-30)– which corresponds to Phase 2 of the MRC framework – involves a pilot study of the holistic care package in one sub-district in Awi zone, which includes research activities to assess the care package's adoption, feasibility, potential effectiveness and fidelity when integrated into government-run health services and across the three diseases, as well as the observable trends in the utilisation and coverage of the care package.

Phase 3 (Months 31-42) – which corresponds to Phases 3 and 4 of the MRC framework – entails the scale-up of the holistic care package of the integrated three disease MMDP embedded into the primary health care facilities, and its evaluation in three selected districts in Awi zone.

Table 1 around here

Sample selection

Awi zone, in which the EnDPoINT study is being conducted, is one of ten zones in the Amhara Region in the North-West of Ethiopia and is divided into twelve districts (or *woredas*). Figure 2 shows the three districts selected for the study: one sub-district (a cluster that includes five villages) in Guagusa Shikudad district for piloting during Phase 2; and three whole districts for the scale-up during Phase 3 (Guagusa Shikudad, Ankesha Guagusa and Guangua). The districts were agreed between the EnDPoINT research team and the Ethiopian FMOH based on: co-endemicity of podoconiosis, LF and leprosy; any previous or ongoing work in the districts; and accessibility. The population of Awi zone is 982,942 (with each district/*woreda* having populations of between around 8000 and 31,500), according to the latest Ethiopian Census in 2007 (52). As for most of Ethiopia, the zone is largely a rural area (around 87.5% of the population (52)); Agew-Awi (59.8%) and Amhara (38.4%) are the two main ethnic groups, and the two most commonly spoken first languages are Amarigna (53.4%) and Agew-Awigna (45.0%). The large majority of the population (94.4%) are Ethiopian Orthodox Christians (52).

Figure 2 around here

For peer review only - http://bmjopen.bmj.com/site/about/guidelines.xhtml

A wide range of stakeholders are involved in EnDPoINT. For the qualitative research activities in all three phases of the study, situational analysis in Phase 1, and the training components in Phases 2 and 3, purposive sampling techniques are being used to identify, approach and recruit key stakeholders based on their role and position. Members of the EnDPoINT Consortium initially contact key stakeholders to ask them to participate in the particular component of the study. Snowballing techniques may also be employed, whereby each of the key stakeholders identified is asked whether they are aware of any other key stakeholders who they think should be included.

For the qualitative work, such as the workshops, key informant interviews and focus groups, participants include members of the EnDPoINT study consortium who have expertise in the provision and/or receipt of care and the context in Ethiopia, current NTD and/or mental health programme implementers (including non-governmental organisations), personnel in the NTD Department of the Ethiopian FMOH, policy-makers, health service planners, health managers and decision-makers, facility-based health workers (e.g. primary health care staff, health officers, nurses), community health workers, community leaders, traditional and religious leaders, members of service user organisations, as well as patients themselves and their families.

For the situational analysis in Phase 1, key health officials and service managers are being approached based on purposive sampling methods if necessary, though most of the information collected for this is being based on secondary data that are available in the public domain.

During the pilot study and scale-up of the care package in Phases 2 and 3, key stakeholders are purposively selected to receive training to implement the various components (interventions) of the care package; this includes staff at the health care organisation level, zonal and district level health office staff, psychiatric nurses, senior health care workers, pharmacy staff, health centre staff (health officers and nurses), community health workers (CHWs) / HEWs, and members of the community (i.e. community leads as facilitators of Community Conversations).

Affected people, i.e. those with lower-limb lymphoedema caused by podoconiosis, LF and leprosy, as well as their families and communities, are being included throughout the study; these are people identified as requiring care for their lymphoedema and/or co-morbid mental ill-health. Patients are identified and recruited into the study based on their case identification within the EnDPoINT programme, i.e. all adult patients (i.e. patients over 18 years of age) within the sub-district in Phase 2 and the three districts during Phase 3 who are identified as having either lymphoedema due to podoconiosis, LF or leprosy will be invited into the study (a few of whom will be selected as case studies). Apart from being beneficiaries of some interventions, patients will also potentially contribute to the delivery of some of the training packages and/or interventions, for example by contributing to training sessions in order to provide a 'lived testimony' of living with the disease, facilitating patient self-help groups, and being actively involved in awareness-raising and stigma reduction activities such as Community Conversations; this is because of growing evidence that social contact elements within interventions, whereby people come into contact with affected patients and hear their stories, are most effective in reducing stigma and discrimination, and can contribute to increased service uptake and provision.

Only adults are being included in the study, i.e. participants are all at least 18 years of age. All components of the EnDPoINT study are being conducted in either Amharic or English, depending on which language is most suitable to the particular participant group. Where necessary, all study

For peer review only - http://bmjopen.bmj.com/site/about/guidelines.xhtml

instruments and materials, including participant information sheets and consent forms, are translated into Amharic, which is the official language of Ethiopia and can be spoken and understood by the majority of people in Ethiopia, including in Awi zone.

Sample size justification

For the qualitative research activities, such as key informant interviews and focus group discussions, the sample size is guided by the number of key stakeholders who are identified to take part. A data saturation approach is being used (i.e. data collection will continue until sufficient information has been obtained or where further data collection fails to generate new/additional themes), which is usual and appropriate for qualitative data collection techniques. For the workshops and training components, the sample size will be established based on the number of people who are identified to be suitable and available. However, the sample size during any workshops and focus groups will not exceed 16 participants, to ensure that all participants have the opportunity to speak.

During Phase 2, all health staff in the selected sub-district are trained. During Phase 3, in the three districts five health workers from each health centre will be trained in the 13 health centres identified through the situational analysis in Phase 1 and they will cascade the training to the remaining 15 staff at each health centre; a total of 150 health extension workers in the three districts will be trained, which is around 10 health professionals across the three districts (there are 4 to 5 health facilities per district). Similarly, all adult patients who are identified within the single sub-district during Phase 2 and within the three whole districts during Phase 3 as having either lymphoedema caused by podoconiosis, LF or leprosy will receive the care interventions included in the care package (or will at least be offered these); we are expecting to identify around 1500-1600 people with lymphoedema due to podoconiosis, leprosy or LF in total across the three districts during Phase 3 based on previous surveys that have been conducted (we do not have numbers available for LF and leprosy).

Key interventions within the care package

The EnDPoINT care package is organised according to three levels of the health care system: health care organisation/coordination, health care facility, and community level. This may include the following interventions, though these will be finalised only after completion of Phase 2 due to the iterative nature of the study:

- Health care organisation/coordination:
    - High-level awareness-raising and mobilisation: participatory workshop for zonal and district health bureau officials, and evidence generation for best practice
    - Programme management, i.e. working in partnership with key health care organisation staff to ensure the necessary structures and budget for the delivery of the care package
    - Capacity-building, i.e. ‘Training of Trainers’ (ToT) for health care coordination staff about lymphoedema MMDP and mental health care provision, and supportive supervision, mentoring and coaching
- Health facility:

For peer review only - http://bmjopen.bmj.com/site/about/guidelines.xhtml

  - Capacity-building for facility-based health centre staff: training on lymphoedema MMDP and mental health care provision, and training on supply chain management for those staff who manage MMDP supplies
  - Awareness-raising and stigma reduction: i) participatory workshop for facility-based staff; ii) posters in health centres, including waiting rooms; iii) health education sessions by health facility staff for attendees of health facilities
  - Case detection, assessment, and treatment initiation by health centre nurses and health officers, including training of patients in self-care, patient counselling and coping skills acquisition, and mental health care
  - Clinical mentoring for facility-based health workers
  - Supportive supervision for facility-based health workers and CHWs
- Community:
  - Capacity-building for CHWs: i) training on lymphoedema MMDP and mental health care provision; ii) Community Conversation (CC) facilitator training
  - Community awareness-raising and stigma reduction: i) awareness-raising workshops for members of the community by CHWs; ii) Community Conversation groups; iii) information dissemination in the community by CHWs (information leaflets, posters in health posts, mass media)
  - Active case detection and referral by CHWs
  - Patient follow-up visits by CHWs
  - Community-based rehabilitation, including home visits, meetings, workshops, patient associations and self-help groups, family support groups, and community mobilisation
  - Supportive supervision for CHWs

In addition, cutting across the three levels of the health care system, there may also be a community advisory group of a wide range of stakeholders across the three levels. It is important to ensure high quality of the health care providers so that they are able to combine physical health, mental health, and psychosocial care.

Procedures

See Table 1 for an overview of the research activities/methods conducted during each of the three phases of the EnDPoINT study.

Development of care package during Phase 1

During Phase 1, a document review of grey literature and a separate systematic review are conducted to inform and guide the development of the holistic care package and strategies for its integration into the routine health care delivery system in Ethiopia. To contribute further to this, a situational analysis / resource assessment is carried out to collect cross-sectional baseline data on factors relevant to the development and integration of the care package in Awi zone in Ethiopia, as well as any resources that are available for this, and potential risk factors. Three 'Theory of Change' (ToC) workshops are conducted to map out the care package's ToC in terms of defining its desired outcomes, indicators, interventions and measurement of the outcomes, which are represented graphically on a

For peer review only - http://bmjopen.bmj.com/site/about/guidelines.xhtml

For peer review only

ToC map along with an accompanying narrative describing the key pathways and assumptions, as well as to encourage stakeholder buy-in to the study (48). Key informant interviews and focus groups are then conducted, to test the feasibility, acceptability and appropriateness of the draft care package, as well as to assess key aspects of the ToC, for example some of the key assumptions identified during the workshops (48). Participants of this qualitative work and the ToC workshops include patients, caregivers and various health providers. The care package and its associated training materials are discussed and finalised following a workshop with members of the EnDPoINT research team and FMOH in Ethiopia. A separate manuscript will be prepared detailing the Phase 1 activities further.

A large focus of the research content during all of the Phase 1 research activities outlined above are the mental health and psychosocial components of the care package, including the stigma reduction elements, as well as how to integrate care across the three diseases and into the routine health care system, as this is where there are gaps in knowledge within the Ethiopian context. In line with this, based on the research activities during Phase 1, already-existing MMDP guidelines for podoconiosis and LF are adapted as part of the project to include leprosy and mental health components, to inform the training materials.

Piloting of care package during Phase 2

During the pilot study in one sub-district in Awi zone, the main training elements and interventions of the care package are tested through a mixed-methods design, to establish their adoption, feasibility, acceptability, fidelity, potential effectiveness, their readiness for scale up, costs of the interventions, and the suitability of the training and training materials. This involves implementing the interventions, including their training components, as outlined in the section on the draft care package above, and evaluating them During the pilot study, the interventions covered by the training are implemented for three months.

Quantitative evaluations of the interventions and associated trainings are conducted at baseline and three-month follow-up through patient surveys with those affected people who are receiving the treatment interventions within the care package, to measure changes in patient outcomes in relation to physical health, mental health/wellbeing, quality of life, stigma and discrimination (including social support), disability, and health care use and costs; surveys with members of the community within which the affected persons live, to assess changes in stigma and 'change of knowledge, attitudes and practice' (KAP); as well as an evaluation of the training through assessment of KAP of health workers receiving the training and their satisfaction with the training. In addition, qualitative assessments are carried out through observation of the interventions being implemented; and key informant interviews and focus group discussions with those stakeholders who received the training and delivered the interventions, as well as the recipients of the interventions (i.e. people affected by podoconiosis, LF and leprosy). Table 2 shows the outcomes that are assessed in Phase 2 together with how these are measured; see also right-hand column in Table 1 for the outcome measurements used. All measurement tools are administered by data collectors rather than being completed by participants themselves, meaning that illiterate participants are not disadvantaged.

The care package, as well as the training materials, may be revised further based on the results of the pilot study (success criteria will be established a priori), and there may be further pilot-testing if major challenges are encountered that will need to be addressed further, before being scaled up in three whole districts.

For peer review only - http://bmjopen.bmj.com/site/about/guidelines.xhtml

Table 2 around here

Scale-up of care package during Phase 3

The scale-up and evaluation of the care package during Phase 3 of the EnDPoINT study will involve implementation of all interventions at all three levels of the health system (health care organisation/coordination, health facility and community level) in three districts in Awi zone. Whilst the exact interventions that will be included in the care package for scale-up will depend on the findings of Phases 1 and 2, the general potential structure of the training for the various interventions included in the care package is depicted in Figure 3.

Figure 3 around here

The scale-up of the care package interventions during Phase 3 will be implemented, followed up and evaluated over a period of one year. Patients will be followed up every month for the first three months and then at 12 months. A mixed-methods approach will be used for the evaluation of the care package. A pre-post design will be employed for quantitative evaluation of the care package, with data collected at baseline (i.e. before training or any of the intervention components commence), and at 3- and 12-month follow-up; data will be collected on coverage, as well as implementation, clinical (physical health, mental health and psychosocial), economic (including total costs of the care package) and social outcomes (see Table 1). The questionnaires for evaluation are the same ones used during the Phase 2 piloting, though these may be refined and improved based on the piloting. A detailed monitoring and evaluation plan for Phase 3 will be developed based on the results in Phase 2.

For the qualitative evaluation of the care package during Phase 3, key informant interviews and focus group discussions will be carried out with a wide range of stakeholders who have been involved in the implementation of the care package (e.g. patients and their families, health facility staff, community health workers, health facility management staff, district-level health staff etc.); these will be carried out at two time points, once towards the beginning of the scale-up period and then again towards the end of the scale-up period. The topic guides and interview questions for the key informant interviews and focus group discussions will be developed once Phase 2 has been completed.

Outcomes to be measured

Tables 1 and 2 detail the outcomes measured during EnDPoINT, as well as the time points and methods that will be used to measure these.

Data analysis plan

Qualitative data (such as from the key informant interviews and focus group discussions) will be analysed using thematic analysis with the assistance of a qualitative software package (NVivo). Validity (dependability) of data will be assessed through the identification of the initial codes by an external experienced qualitative researcher and agreeing on the framework in which the data will be

For peer review only - http://bmjopen.bmj.com/site/about/guidelines.xhtml

For peer review only

presented, presentation of initial findings to experienced academics to assess the plausibility, and including single counting of identified events/phenomena. Validity assurance will also involve checking of alignment of research questions, interview guides, sampling procedures etc. For quantitative data collected during Phases 2 and 3, descriptive analyses (such as simple counts and frequencies or means and standard deviations) will be used, as well as the % change before and after the implementation of the interventions, together with 95% confidence intervals. A detailed data analysis plan will be produced for the evaluation of the care package during Phases 2 and 3. The results from the various research activities throughout the study will be triangulated to develop and finalise the holistic care package and strategies for its integration into the routine health care delivery system in Awi zone in Ethiopia.

Patient and public involvement

Patients and their communities are being fully engaged in the research process throughout the entire EnDPoINT project. EnDPoINT is being supported by a Consortium, which includes patient representatives alongside researchers, health professionals, FMOH staff and other stakeholders, whose remit is to provide input, guidance and advice throughout the programme of research. Patients and community members are partnering with the EnDPoINT study team for the design of the study and care package. This includes the development and implementation of the interventions; the development of materials, such as training materials; the sharing and identification of findings; and the identification of appropriate channels and venues for the dissemination of results. Affected people and their families and communities are included within the workshops and qualitative aspects of the work, such as the key informant interviews and focus group discussions. The situational analysis ensures that the interventions fit in with the local context and build on the strengths and assets of the community. Where possible, members of the local community are being employed to collect data, to facilitate capacity-building of community members. The EnDPoINT Consortium will contribute to various outputs for dissemination. Patients and community members are therefore being involved in the entire study process, both as co-researchers and as participants of the study.

Economic analysis

We aim to cost the holistic package of physical health, mental health and psychosocial care interventions for people with lower-limb lymphoedema caused by podoconiosis, LF or leprosy, and the integration of this package into the routine health care delivery system (Phases 2 and 3), thereby ensuring that the care package represents value for money. Intervention activities will be costed at three levels:

1. Health care organisation/coordination level: awareness-raising activities, programme management and training health care organisation staff;
2. Health care facility level: awareness-raising activities, case detection, treatment, staff training in health education for patient physical health self-care, mental health and psychosocial support;
3. Community level: awareness-raising activities, training and supervision of CHWs, active case detection by CHWs, providing community-based health care and rehabilitation.

For peer review only - http://bmjopen.bmj.com/site/about/guidelines.xhtml

For peer review only

The main cost components will be identified in the pilot study (Phase 2); cost data will be collected during the scale-up of the care package intervention (Phase 3). A micro-costing approach will include a bottom-up construction of costs associated with setting-up and delivering the package. The costs of training healthcare professionals will include: time spent in preparing and attending the training sessions, room rental, training materials, travel expenses, and administration costs. The costs of delivering the healthcare package will include: costs of lymphoedema treatment supplies (e.g. soap, ointment, bandages, gloves, antiseptics), infrastructure costs, and additional staff salaries/time. Costs will be collected from financial records and interviews with programme managers. We will also collect data on the socio-economic characteristics of study participants, housing, economic activities, use of health care services and out-of-pocket expenses related to the lower limb lymphoedema. Direct costs will cover contacts with health care professionals, pharmacists and traditional healers, medication and hospitalisations with respect to the conditions. Indirect costs will include additional costs to the individuals and their families (e.g. travel to hospital, accommodation, food and borrowing money from family/relatives/community). Economic data will be collected using a purpose-designed questionnaire.

ETHICS AND DISSEMINATION

Ethics

Ethical approval has already been obtained from the Brighton and Sussex Medical School Research Governance and Ethics Committee (BSMS RGEC) in the UK, as well as the Institutional Review Board of the College of Health Sciences at Addis Ababa University in Ethiopia.

All people who are participating in the research (all over 18 years old) are given a participant information sheet (outlining the study's research aims, procedures, and potential risks and benefits) and are required to give their written consent to take part (or verbal consent with a witness declaration by the interviewer where a person is illiterate); if a person does not consent to take part, that person is excluded. However, those people who are involved purely in the implementation of the care package, for example those who are receiving training on self-care for lymphoedema or who are taking part in a stakeholder meeting, are not required to give their informed consent, though they are not obliged to be involved. All participants are able to withdraw from the study at any time without having to give a reason or any detrimental consequences, for instance in regard to their care, treatment or employment, until data have been aggregated.

Confidentiality and anonymity of data is ensured throughout the study process, from data collection to data storage, analysis and publication. Structured, multi-level precautions are taken to safeguard the confidential nature of the information gathered, and to ensure the anonymity of participants, for example by using personal identifiers instead of names, with identifying data kept separately from the identifying codes used on the data collection sheets and databases. Only anonymised data will be used for data analyses. All data are being stored in a secure OneDrive folder, which can only be accessed by members of the team for whom access to the data are essential.

For peer review only - http://bmjopen.bmj.com/site/about/guidelines.xhtml
For peer review only

Dissemination

An EnDPoINT publication plan has already been developed, which includes several planned articles for publication in scientific journals. It is likely that the results of the study will also be shared through various other forums, including to the larger scientific community through conferences, but also to policymakers and health care staff, and other participants of the study. This will likely be in the form of a workshop at the end of the study and/or through the distribution of a summary document and/or policy brief(s) with an overview of the main results of the study.

For peer review only

Figure 1: 'Theory of Change' within the MRC framework on complex interventions (taken unchanged from de Silva et al 2014 (48)) (in white: taken directly from MRC framework; in blue: added elements on ToC)

Figure 2: Implementation districts for the EnDPoINT study

Figure 3: Potential training structure within the EnDPoINT study

For peer review only - http://bmjopen.bmj.com/site/about/guidelines.xhtml

References

1. Deribe K, Cano J, Giorgi E, et al.; Estimating the number of cases of podoconiosis in Ethiopia using geostatistical methods. *Wellcome Open Research* 2017;**2**:78.
2. International Federation of Anti-Leprosy Associations (ILEP). https://www.ilepfederation.org/ (Date Accessed 2020 Accessed, date last accessed)
3. Rebollo M, Sime H, Assefa A, et al.; Shrinking the Lymphatic Filariasis Map of Ethiopia: Reassessing the Population at Risk through Nationwide Mapping. *PloS NTDs* 2015;**9**(11):e0004172. doi: 10.1371/journal.pntd.000417.
4. Sime H, Gass K, Mekasha S, et al.; Results of a confirmatory mapping tool for Lymphatic filariasis endemicity classification in areas where transmission was uncertain in Ethiopia. *PloS NTDs* 2018;**12**(3):e0006325.
5. Kebede B, Martindale S, Mengistu B, et al.; Integrated morbidity mapping of lymphatic filariasis and podoconiosis cases in 20 co-endemic districts of Ethiopia. *PloS NTDs* 2018;**12**(7):e0006491. doi: doi: 10.1371/journal.pntd.0006491.
6. Deribe K, Brooker S, Pullan R, et al.; Epidemiology and Individual, Household and Geographical Risk Factors of Podoconiosis in Ethiopia: Results from the First Nationwide Mapping. *Am. J. Trop. Med. Hyg* 2015;**92**(1):148-158.
7. Ottesen E, Hooper P, Bradley M, et al.; The Global Programme to Eliminate Lymphatic Filariasis: Health Impact after 8 Years. *Plos NTDs* 2008;**2**(10):e317. doi: https://doi.org/10.1371/journal.pntd.0000317.
8. World Health Organization (WHO); Weekly epidemiological record. In: WHOs (ed). Geneva: WHO, 2013, 365-380.
9. Centers for Disease Control and Prevention (CDC). *World Leprosy Day: Bust the Myths, Learn the Facts*. https://www.cdc.gov/features/world-leprosy-day/index.html (Date Accessed 2018 Accessed, date last accessed)
10. Tekola A, Adeyemo A, Finan C, et al.; HLA class II locus and susceptibility to podoconiosis. *New England Journal of Medicine* 2012;**366**:1200-1208.
11. Bekri W, Gebre S, Mengiste A, et al.; Delay in presentation and start of treatment in leprosy patients: a case-control study of disabled and non-disabled patients in three different settings in Ethiopia. *International Journal of Leprosy* 1998;**66**(1):1-9.
12. Mousley E, Deribe K, Tamiru A, et al.; Mental distress and podoconiosis in Northern Ethiopia: a comparative cross-sectional study. *International Health* 2014. doi: 10.1093/inthealth/ihu043.
13. Bartlett J, Deribe K, Tamiru A, et al.; Depression and disability in people with podoconiosis: a comparative cross-sectional study in rural Northern Ethiopia. *International Health* 2016. doi: doi:10.1093/inthealth/ihv037.
14. Semrau M, Davey G, Beng A, et al.; Depressive symptoms amongst people with podoconiosis and lower limb lymphoedema of other cause in Cameroon: A cross-sectional study. *Tropical Medicine and Infectious Disease.* 2019;**4**(3):E102. doi: doi:10.3390/tropicalmed4030102.
15. Hofstraat K, van Brakel W; Social stigma towards neglected tropical diseases: a systematic review. *International Health* 2016;**8**(s1):i53-i70.
16. Tora A, Davey G, Tadele G; A Qualitative Study on Stigma and Coping Strategies of Patients with Podoconiosis in Wolaita Zone, Southern Ethiopia. *International Health* 2011;**3**(3):176-181.
17. Tora A, Franklin H, Deribe K, et al.; Extent of podoconiosis-related stigma in Wolaita Zone, Southern Ethiopia: a cross-sectional study. *Springer Plus* 2014;**3**:647.
18. Person B, Bartholomew L, Gyapong M, et al.; Health-related stigma among women with lymphatic filariasis from the Dominican Republic and Ghana *Social Science & Medicine* 2009;**68**(1):30-38.
19. Tekola F, HaileMariam D, Davey G; Economic costs of endemic non-filarial elephantiasis in Wolaita Zone, Ethiopia. *Tropical Medicine & International Health* 2006;**11**(7):1136-1144.
20. Perera M, Whitehead M, Molyneux D, et al.; Neglected Patients with a Neglected Disease? A Qualitative Study of Lymphatic Filariasis. *PLoS Neglected Tropical Diseases* 2007;**1**(2):128.

For peer review only - http://bmjopen.bmj.com/site/about/guidelines.xhtml

21. Martindale S, Mkwanda S, Smith E, et al.; Quantifying the physical and socio-economic burden of filarial lymphoedema in Chikwawa District, Malawi. *Transactions of the Royal Society of Tropical Medicine & Hygiene* 2014;**108**(12):659-667.
22. Slanton M, Yamauchi M, Mkwanda S, et al.; Measuring the physical and economic impact of filarial lymphoedema in Chikwawa district, Malawi: a case-control study. *Infect Dis Poverty* 2017;**6**(1):28. doi: 10.1186/s40249-017-0241-2.
23. Deribe K, Negussu N, Newport M, et al.; The health and economic burden of podoconiosis in Ethiopia. *Trans R Soc Trop Med Hyg* in press.
24. Hotez P, Fenwick A, Savioli L, et al.; Rescuing the "bottom billion" through neglected tropical disease control. *Lancet* 2009;**373**:1570-1576.
25. Ton T, Mackenzie C, Molyneux D; The burden of mental health in LF. *Infect Dis Poverty* 2015;**4**:34.
26. Ton T, Mackenzie C, Molyneux D; The burden of mental health in LF. *Infect Dis Poverty* 2015;**4**:34.
27. Tora A, Davey G, Tadele G; Factors related to discontinued clinic attendance by patients with podoconiosis in southern ethiopia: a qualitative study. *BMC Public Health* 2012;**12**:902.
28. Tsegaye G, Woubie M, Degu G, et al.; Barriers to access and re-attendance for treatment of podoconiosis: a qualitative study in Northern Ethiopia. *International Health* 2014.
29. Hofstraat K, van Brakel W; Social stigma towards neglected tropical diseases: a systematic review. *Int Health* 2016;**8**(suppl 1):i53-i70.
30. Tora A, Davey G, Tadele G; A qualitative study on stigma and coping strategies of patients with podoconiosis in Wolaita zone, Southern Ethiopia. *Int Health* 2011;**3**:176-181.
31. Molla Y, Tomczyk S, Amberbir T, et al.; Patients' perceptions of podoconiosis causes, prevention and consequences in East and West Gojam, Northern Ethiopia. *BMC Public Health* 2012;**12**(828).
32. Yakob B, Deribe K, Davey G; High levels of misconceptions and stigma in a community highly endemic for podoconiosis in southern Ethiopia. *Trans Roy Soc Trop Med Hyg* 2008;**102**(5):439–444.
33. Ayode D, McBride C, de Heer H, et al.; The association of beliefs about heredity with preventive and interpersonal behaviors in communities affected by podoconiosis in rural Ethiopia. *Am J Trop Med Hyg* 2012;**87**(4):623-30.
34. World Health Organization (WHO). *Lymphatic filariasis - Policy.* https://www.who.int/lymphatic_filariasis/policy/en/ (Date Accessed 2020 Accessed, date last accessed)
35. World Health Organization (WHO). *Leprosy elimination - Elimination of leprosy FAQ.* https://www.who.int/lep/strategy/faqs/en/ (Date Accessed 2020 Accessed, date last accessed)
36. Deribe K, Meribo K, Gebre T, et al.; The burden of neglected tropical diseases in Ethiopia, and opportunities for integrated control and elimination. *Parasites and Vectors* 2012;**5**:240.
37. Obindo J, Abdulmalik J, Nwefoh E, et al.; Prevalence of depression and associated clinical and socio-demographic factors in people living with lymphatic filariasis in Plateau State, Nigeria. *PLOS Neglected Tropical Diseases* 2017;**11**(6):e0005567.
38. Demyttenaere K, Bruffaerts R, Posada-Villa J, et al.; Prevalence, severity, and unmet need for treatment of mental disorders in the World Health Organization World Mental Health Surveys. *JAMA* 2004;**291**:2581–90.
39. Prochazka M, Timothy J, Pullan R, et al.; Buruli ulcer and leprosy, they are intertwined": Patient experiences of integrated case management of skin neglected tropical diseases in Liberia. *PLoS Negl Trop Dis* 2020;**14**(2):e0008030.
40. Koffi A, Yao T, Barogui Y, et al.; Integrated approach in the control and management of skin neglected tropical diseases in three health districts of Côte d'Ivoire. *BMC Public Health* 2020;**20**(1):517.
41. Deribe K, Wanji S, Shafi O, et al.; The feasibility of eliminating podoconiosis. *Bull World Health Organ* 2015;**93**(10):712-8.

For peer review only - http://bmjopen.bmj.com/site/about/guidelines.xhtml

42. Ministry of Health FDRoE; National Masterplan for Neglected Tropical Diseases (NTDs). Addis Ababa: Ministry of Health FDRoE, 2013, 1-56.
43. World Health Organization (WHO). *Sixty ninth World Health Assembly adopts resolution on mycetoma*. http://www.who.int/neglected_diseases/news/WHA_69_adopts_mycetoma_resolution/en/ (Date Accessed 2016 Accessed, date last accessed)
44. McBride C, Price C, Ayode D, et al.; A cluster randomised intervention trial to promote shoe use by children at high risk for podoconiosis. *Int J Health Sci Res* 2015;**5**(6):518-528.
45. Peters D, Tran N, Adam T; Implementation research in health - A practical guide. Geneva: World Health Organization, 2013.
46. Craig P, Dieppe P, Macintyre S, et al.; Developing and evaluating complex interventions: the new Medical Research Council guidance. *British Medical Journal* 2008;**337**:a1655. doi: 10.1136/bmj.a1655.
47. Medical Research Council (MRC). *Developing and evaluation complex interventions: new guidance*. https://www.mrc.ac.uk/documents/pdf/complex-interventions-guidance/ (Date Accessed, date last accessed)
48. De Silva M, Breuer E, Lee L, et al.; Theory of Change: a theory-driven approach to enhance the Medical Research Council's framework for complex interventions. *Trials* 2014;**15**:267.
49. Breuer E, de Silva M, Fekadu A, et al.; Using workshops to develop theories of change in five low and middle income countries: lessons from the programme for improving mental health car (PRIME). *International Journal of Mental Health Systems* 2014;**8**:15.
50. Craig P, Dieppe P, Macintyre S, et al.; Developing and evaluating complex interventions: the new Medical Research Council guidance. *BMJ* 2008;**337**:a1655.
51. Negussie H, Molla M, Ngari M, et al.; Lymphoedema management to prevent acute dermatolymphangioadenitis in podoconiosis in northern Ethiopia (GoLBeT): a pragmatic randomised controlled trial. *Lancet Global Health* 2018;**6**:e795-e803.
52. Office of the Population Census Commission (OPCC); The 2007 Population and Housing Census of Ethiopia. 2007.

For peer review only - http://bmjopen.bmj.com/site/about/guidelines.xhtml

Authors' contributions

All authors contributed to the manuscript and have accepted the final version. MS led the writing of the manuscript. GD and AF are the Principal Investigators of the EnDPoINT study. OAA, AM, AT, MK, SAB and NH are part of the core EnDPoINT research team; OAA, AM, AT and MK are responsible for the implementation of the study in Ethiopia; SAB leads the statistical elements of the study; and NH leads the economic aspects. KD produced two of the figures in the manuscript, and is a member of the EnDPoINT Research Consortium, as are LAK-H, HM, HBT, HB, DH and NN. All members of the EnDPoINT Research Consortium have actively contributed to the inception of the study and continuous deliberations since, in relation to conceptual and methodological approaches and implementation.

Acknowledgements

We thank the other members of the EnDPoINT Research Consortium for their input into the study, as follows: Ms Tsige Amberbir, Ms Tanny Hagens, Mr Mossie Tamiru, Mr Tadesse Tesfaye, Dr Seifu Tirfie, and Dr Abebayehu Tora. We also thank Ms Clare Callow for her project management of the NIHR grant through which EnDPoINT is funded, as well as Mr Tesfaye Asefa, Ms Bethelhem Fekadu, Ms Grit Gansch, Ms Samrawit Ketema for their support with the administrative aspects of the study. Our sincerest thanks to all participants of the EnDPoINT study.

Funding

This research was commissioned by the National Institute for Health Research (NIHR) Global Health Research Unit on NTDs at BSMS using Official Development Assistance (ODA) funding. The views expressed in this publication are those of the author(s) and not necessarily those of the NIHR or the Department of Health and Social Care. KD is supported by the Wellcome Trust as part of his International Intermediate Fellowship [grant number 201900].

Competing interests

The authors have no competing interests to declare.

For peer review only

For peer review only - http://bmjopen.bmj.com/site/about/guidelines.xhtml

Table 1 Objectives, research questions, research activities/methods and outcome measures for each of the three phases within the EnDPoINT study

	Objectives	Research questions	Research activities/methods	Outcome measures
**Phase 1 – Development of care package**	• Finalise a comprehensive package of holistic physical health, mental health and psychosocial care for people with podoconiosis, LF and leprosy<br>• Learn lessons about integration from implementers and beneficiaries of care to date<br>• Develop strategies for integrating and evaluating the holistic care package into the routine health care delivery system in selected districts in Awi zone in Ethiopia, and integrating care across the three diseases	• What are the key elements that constitute optimal physical health, mental health and psychosocial care for patients with podoconiosis, LF and leprosy?<br>• What strategies need to be developed to facilitate integration of the holistic care package into the routine health care delivery system and across the three diseases?<br>• What are the critical contextual factors (including drivers and barriers) that influence the process of integration of the - holistic care package into government-run health services and across the three diseases?<br>• What are the key features of the intervention that influence the manner of integration into the health care system and across the three diseases?<br>• Who are the key health system actors that have a stake in the integration of the care package into the government-run health services, and what coordination	• *Document review* of grey literature, including existing national NTD guidelines, other relevant documents on care provision for NTDs and/or mental health, study reports and programme documents, to inform and guide the development of the care package.<br>• *Systematic review* of publications in scientific journals, on the functional/disability, mental health and psychosocial outcomes secondary to podoconiosis, LF and leprosy, to complement the document review.<br>• *Situational analysis / resource mapping*, to collect cross-sectional baseline data on contextual factors relevant to the development, implementation and integration of the care package, as well as to identify any resources available for this, and potential risk factors.<br>• *Three 'Theory of Change' (ToC) workshops* with members of the research team and key stakeholders, to identify and establish the key causal pathways between the desired outcomes, interventions, assumptions, indicators and measurement of the outcomes for the care package (represented visually through a ToC map), as well as to encourage stakeholder buy-in to the study (48).<br>• *Key informant interviews and focus group discussions* with stakeholders, to assess the draft care package's feasibility (i.e. the extent to which the intervention can be carried out within the	Qualitatively assessed implementation outcomes, including feasibility, acceptability and appropriateness.

For peer review only - http://bmjopen.bmj.com/site/about/guidelines.xhtml

		and capacity building needs exist?<br>• Is the draft care package feasible, acceptable and appropriate in terms of its integration into government-run health services?	routine health system), acceptability (i.e. the perception among stakeholders that the care package is agreeable), and appropriateness (i.e. the perceived fit or relevance of the care package to key stakeholders), and to assess key aspects of the ToC (for example, assumptions made).<br>• *Workshop* with key stakeholders to discuss the draft *care plan and training materials* that will be adapted.<br>• Informed by the above steps, *develop a comprehensive care package* consisting of interventions at the healthcare organisation/coordination, health facility and community level.	
**Phase 2 – Piloting of care package in one sub-district**	• Implement and evaluate the care package in one sub-district in Awi zone in Ethiopia<br>• Develop a monitoring and evaluation plan for the subsequent scale-up of implementation of the care package	The research questions outlined for Phase 1 are sustained in Phase 2. Additional research questions during Phase 2 are:<br>• Is the holistic care package adoptable, feasible, potentially effective, and of high fidelity when integrated into government-run health services and across the three diseases?<br>• What are the observable trends in the utilisation and coverage of the care package?	• *Pilot study of the care package in one sub-district* in Awi zone, to assess its adoption (i.e. the intention of trying to employ the care package), feasibility, acceptability, fidelity (i.e. the degree to which the care package was implemented as designed), effectiveness (i.e. the impact of the care package as delivered on individual patient outcomes), costing of the care package, its readiness for scale-up, and the suitability of the training and training materials. This is achieved through:<br>○ Observation<br>○ Key informant interviews and/or focus group discussions with key stakeholders such as people who received the training and/or those delivered or received the	• Qualitatively assessed implementation outcomes, including adoption, feasibility, acceptability and fidelity.<br>• Effectiveness of care package interventions assessed at baseline and 3-month follow-up, indicated by number of cases identified and treated, and patient-level outcomes (pre-post) through structured questionnaires, including MMDP assessment, swelling circumference, frequency of acute attacks, stage and grade of affected limb, depression

For peer review only - http://bmjopen.bmj.com/site/about/guidelines.xhtml

			interventions; cost data will be collected from programme managers.<br>○ Before-and-after (pre-post) collection of quantitative data, including number of cases identified and treated (and whether there were any differences in the way these were identified), patient-level outcomes, and for the training 'change of knowledge, attitudes and practice' (KAP) and satisfaction questionnaires<br>• *Workshop* with the NTD Department of the Ethiopian FMOH<br>• Based on the pilot study and workshop, *development of a protocol to evaluate scale-up* of integration of the holistic care package across the three diseases into the state health system in three districts in Awi zone in Ethiopia.	(measured through the Patient Health Questionnaire, PHQ-9), suicidal ideation (CIDI), alcohol use (FAST), quality of life (Dermatology Quality of Life Index, DQLI), disability (WHODAS 2.0), internalised stigma (ISRL), discrimination (DISC-12), social distance (Social Distance Scale, SDS), social support (OSLO 3), happiness index, and explanatory models.<br>• Training evaluations, measured by questionnaires on 'change of knowledge, attitudes and practice' (KAP) (immediately before and after training), and mixed-methods satisfaction data (immediately after training).<br>• Cost of interventions (economic outcomes).
**Phase 3 – Scale-up and evaluation of care package in three districts**	• Scale up the holistic care package across the three diseases into government-run health services in three districts in Awi zone in Ethiopia based on the findings from the pilot study in the single sub-	• What are the critical factors (including drivers and barriers) that influence the process of scaling-up the care package, and that ensure its effectiveness, sustainability, quality and coverage?<br>• How does the context interact with the intervention to	• Implementation of the interventions that are included in the care package and that were developed and piloted during Phases 1 and 2 of the study. These will include interventions at the health care organisation/coordination, health facility and community levels.<br>• Evaluation of care package through mixed methodologies, i.e.:	The exact evaluation plan will be developed and finalised following Phases 1 and 2, but will include evaluation of the care package in regards to coverage (i.e. the degree to which patients in the selected districts actually received the care package), as well as implementation outcomes (including acceptability),

For peer review only - http://bmjopen.bmj.com/site/about/guidelines.xhtml

	district (during Phase 2)<br>• Evaluate the scale-up of the care package<br>• Conduct analysis of the intervention costs during the scale-up	influence the effectiveness of integration, i.e. how do these elements fare in the different contexts presented by the three districts?<br>• Does the care package result in improved outcomes for patients with podoconiosis, LF and leprosy, including clinical (physical health, mental health and psychosocial), economic and social outcomes?<br>• What is the economic impact of the care package?	○ Before-and-after (pre-post) collection of quantitative data, including number of cases identified and treated (and whether there were any differences in the way these were identified), patient-level outcomes, and for the training 'change of knowledge, attitudes and practice' (KAP) and satisfaction questionnaires.<br>○ Key informant interviews and/or focus group discussions with key stakeholders such as people who received the training and/or those delivered or received the interventions; cost data will be collected from programme managers.	clinical (physical health, mental health and psychosocial), economic and social outcomes, similar to the evaluation during the pilot study during Phase 2 (see row above and Table 2). Evaluation data will be collected at baseline, and at 3- and 12-month follow-up.

For peer review only - http://bmjopen.bmj.com/site/about/guidelines.xhtml

Table 2 Outcomes and their measures during Phases 2 and 3 of the EnDPoINT study

**Type of outcomes**	**Specific outcome**	**Outcome measures**
Implementation outcomes	Adoption	Qualitatively (KIIs/FGDs, observation)
	Feasibility	Qualitatively (KIIs/FGDs, observation); cost of training/interventions; number of cases identified, assessed and treated
	Acceptability (by providers and patients)	Qualitatively (KIIs/FGDs, observation)
	Appropriateness	Qualitatively (KIIs/FGDs, observation)
	Fidelity	Qualitatively (KIIs/FGDs, observation)
	Readiness for scale-up	Qualitatively (KIIs/FGDs, observation)
	Economic characteristics of study participants	Purposely-designed questionnaire
	Resource use associated with delivering the care package	Project financial records and interviews with project manager(s)
Effectiveness (patient level)	MMDP assessment	Swelling circumference (physical measurements); frequency of acute attacks (patient self-report); stage/grade of affected limb (physical assessment); signs of infection (physical assessment); wounds (physical assessment); nodules (physical assessment)
	Depression	Patient Health Questionnaire (PHQ-9)
	Suicidal ideation and action	CIDI questions
	Alcohol use	Fast alcohol screening test (FAST)
	Quality of life	Dermatology Life Quality Index (DLQI)
	Disability	WHO Disability Assessment Schedule 2.0 (WHODAS 2.0)
	Internalised stigma	Internalised stigma related to lymphoedema (ISRL)

For peer review only - http://bmjopen.bmj.com/site/about/guidelines.xhtml

	Discrimination	Discrimination and Stigma Scale (DISC-12)
	Social support	Social support scale (OSLO-3)
	Happiness	Happiness index
	Use of primary care/cost	Purposely-designed questionnaire
	Use of hospital care/cost	Purposely-designed questionnaire
	Use of medication/cost	Purposely-designed questionnaire
	Personal expenses	Purposely-designed questionnaire
	Days off work due to illness/ cost	Purposely-designed questionnaire
Effectiveness (community level)	Coverage	Number of cases identified, assessed and treated; proportion of cases detected who are then treated; number of patients reached with MMDP supplies; number of patients who have received mental health care; contact coverage
	KAP lymphoedema	Purposely-designed questionnaire
	Social distance	Social Distance Scale (SDS)
Effectiveness (facility level)	KAP lymphoedema	Purposely-designed questionnaire
	KAP mental health	Purposely-designed questionnaire
Other outcomes	Suitability of training and training materials	Qualitatively (KIIs/FGDs, observation)
	Satisfaction with training	Purposely-designed questionnaire; qualitatively (KIIs/FGDs, observation)

KIIs: key informant interviews; FGDs: focus group discussions; KAP: knowledge, attitudes and practice

For peer review only

For peer review only - http://bmjopen.bmj.com/site/about/guidelines.xhtml

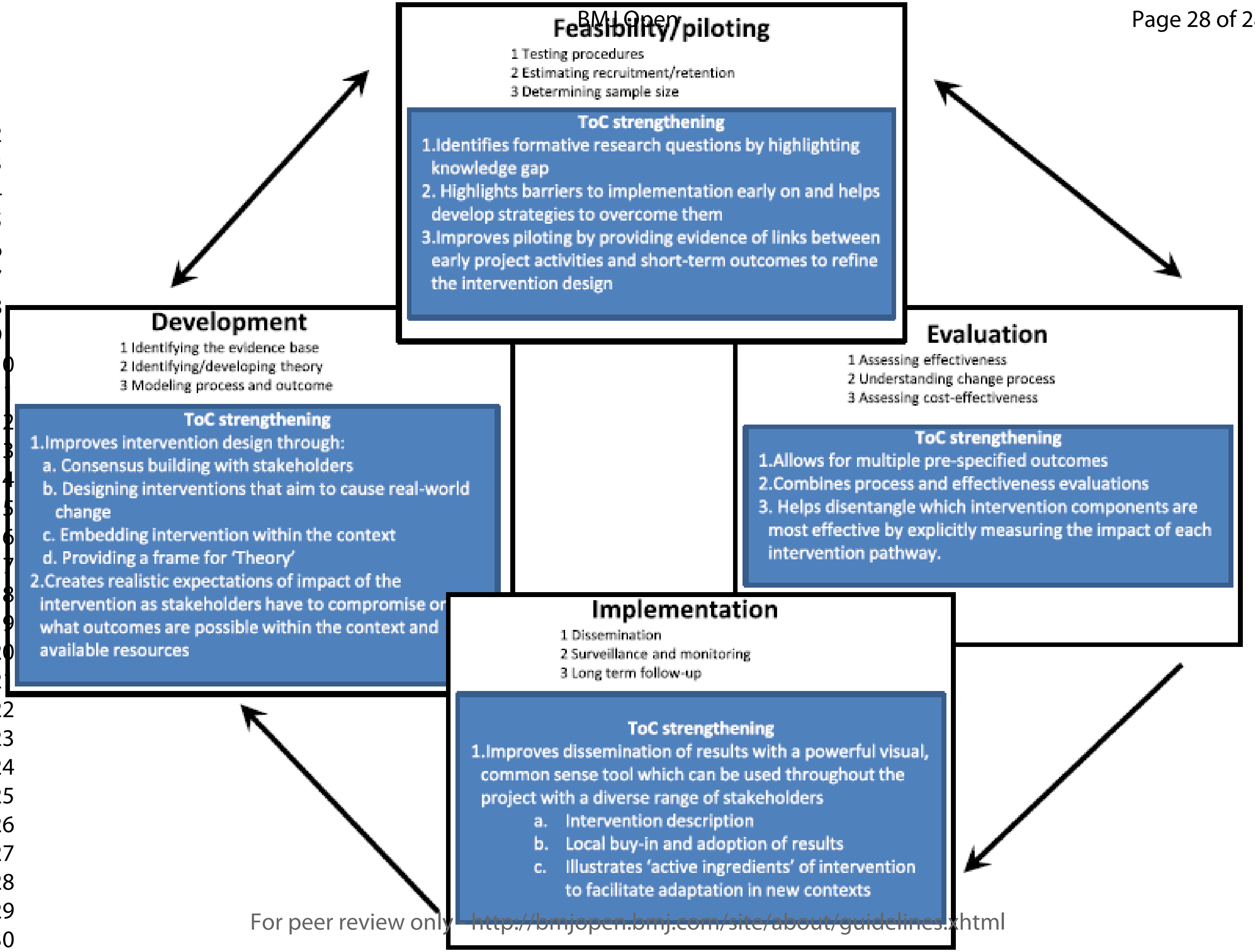

For peer review only - http://bmjopen.bmj.com/site/about/guidelines.xhtml

Figure 2 How Theory of Change can be used to strengthen the MRC framework. Adapted from Craig *et al.* [1].

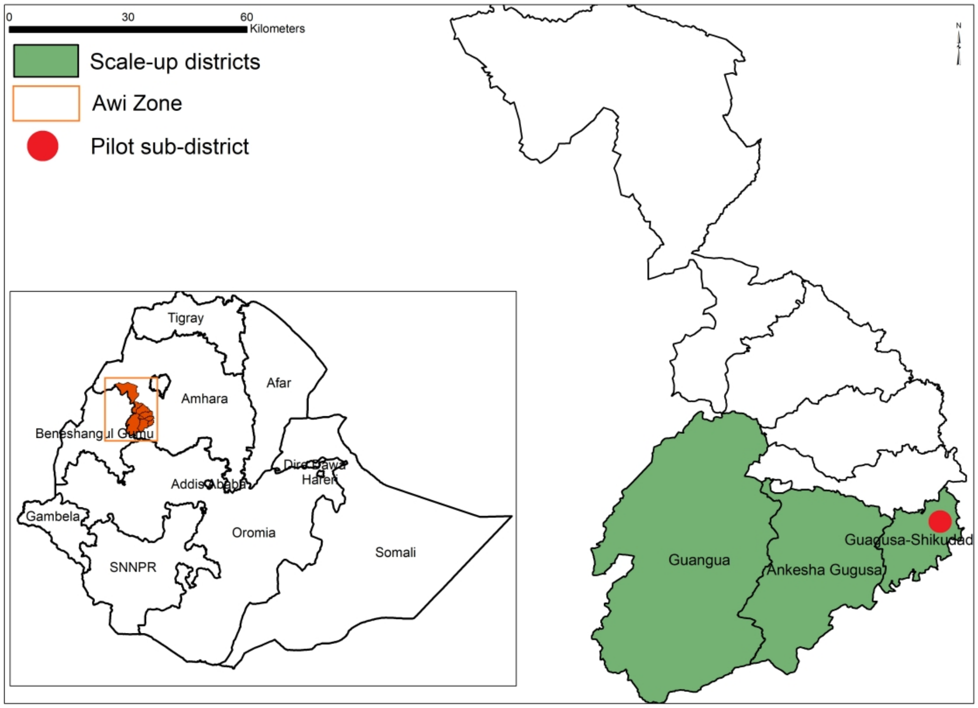

Figure 2: Implementation districts for the EnDPoINT study

419x297mm (300 x 300 DPI)

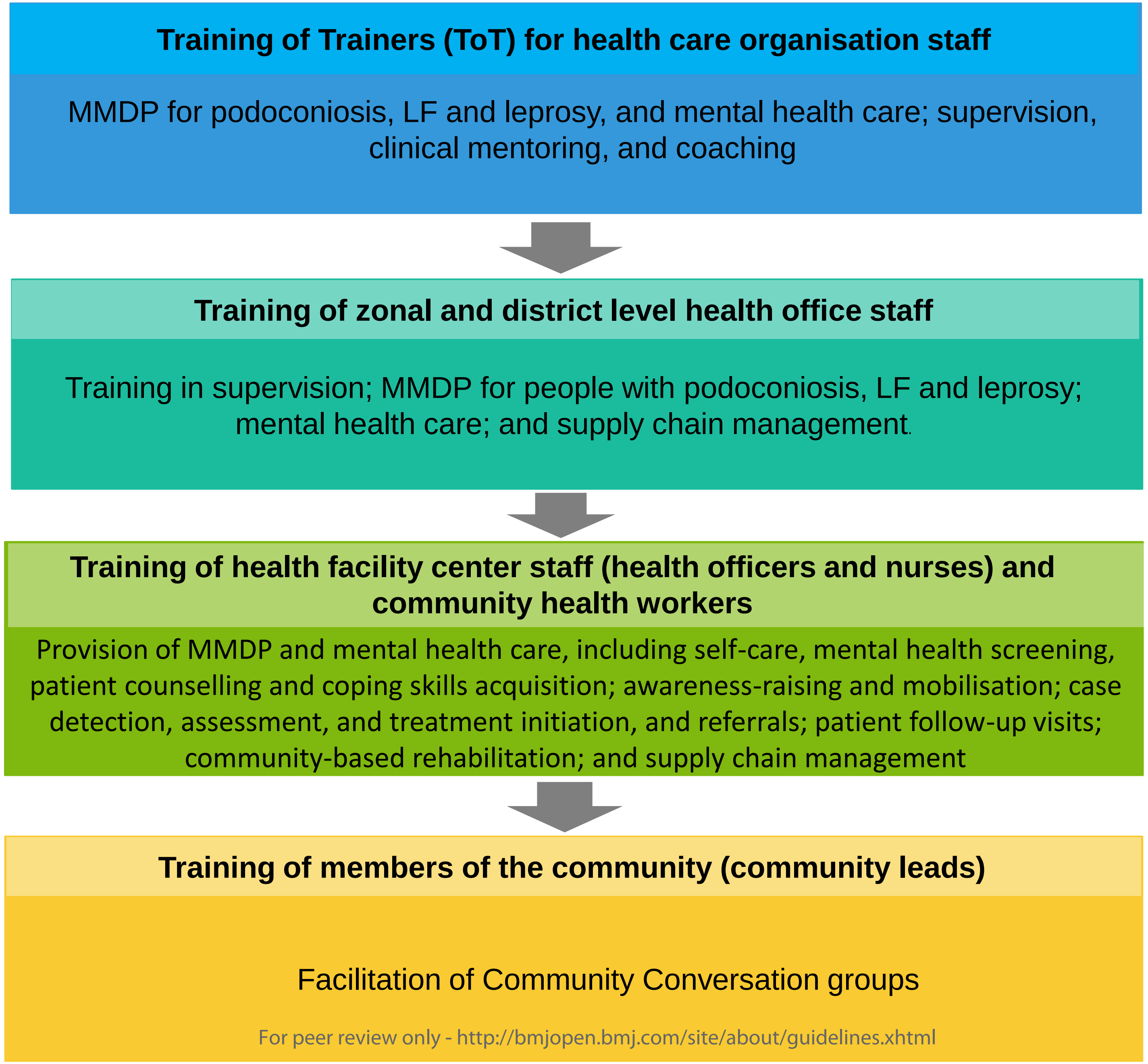
Training of Trainers (ToT) for health care organisation staff
MMDP for podoconiosis, LF and leprosy, and mental health care; supervision, clinical mentoring, and coaching
Training of zonal and district level health office staff
Training in supervision; MMDP for people with podoconiosis, LF and leprosy; mental health care; and supply chain management.
Training of health facility center staff (health officers and nurses) and community health workers
Provision of MMDP and mental health care, including self-care, mental health screening, patient counselling and coping skills acquisition; awareness-raising and mobilisation; case detection, assessment, and treatment initiation, and referrals; patient follow-up visits; community-based rehabilitation; and supply chain management
Training of members of the community (community leads)
Facilitation of Community Conversation groups
